# Influence of Graphene Oxide and Palladium Incorporation on Hydrothermally Grown Hydroxyapatite Coatings on β-Ti30Zr5Mo Alloy: Surface Chemistry, Corrosion and Biological Performance

**DOI:** 10.3390/ma19194092

**Published:** 2026-09-24

**Authors:** Oktay Yigit, Selçuk Karataş, Burak Dikici, Carl J. Boehlert, Jonathan Hardy, Xiaoli Zhao, Mitsuo Niinomi

**Affiliations:** 1 Department of Metallurgical and Materials Engineering, Faculty of Technology, Firat University, Elazig 23119, Türkiye; oktayyigit2561@gmail.com; 2 Department of Metallurgical and Materials Engineering, Faculty of Engineering, Firat University, Elazig 23119, Türkiye; 3 Department of Mechanical Engineering, Ataturk University, Erzurum 25240, Türkiye; burakdikici@atauni.edu.tr; 4 Department of Chemical Engineering and Materials Science, Michigan State University, East Lansing, MI 48824, USA; boehlert@egr.msu.edu; 5 Department of Microbiology and Molecular Genetics, Michigan State University, East Lansing, MI 48824, USA; hardyjon@msu.edu; 6 School of Material Science and Engineering, Northeastern University, Shenyang 110819, China; zhaoxl@smm.neu.edu.cn; 7 Institute for Materials Research, Tohoku University, Sendai 980-8577, Japan; mitsuo.niinomi.b6@tohoku.ac.jp; 8 Titanium Research Center, Institute of Light Metals, University of Toyama, Toyama 933-8588, Japan

**Keywords:** hydroxyapatite coating, graphene oxide, palladium, β-type titanium alloy, corrosion resistance, cellular response

## Abstract

Hydroxyapatite coatings containing graphene oxide and palladium were hydrothermally deposited on a β-type Ti30Zr5Mo alloy in order to establish how the organization of graphene oxide and the incorporation of palladium influence the structural, electrochemical, and biological performance of the coating system. Six compositions—hydroxyapatite, hydroxyapatite with 1 wt% palladium, hydroxyapatite with 2 wt% palladium, hydroxyapatite/graphene oxide, and the corresponding graphene oxide composites containing 1 and 2 wt% palladium—were compared using X-ray diffraction, Fourier-transform infrared spectroscopy, Raman spectroscopy, X-ray photoelectron spectroscopy, scanning electron microscopy combined with energy-dispersive X-ray spectroscopy, electrochemical impedance spectroscopy, potentiodynamic polarization, cell-density analysis with A7R5 cells, and bioluminescence imaging of *Staphylococcus aureus* Xen36. The apatite framework was retained in every composition, and Raman spectroscopy confirmed that the carbon phase derived from graphene oxide survived hydrothermal processing. X-ray photoelectron spectroscopy of the graphene oxide coating containing 2 wt% palladium revealed oxygenated carbon environments and predominantly oxidized, oxygen-coordinated palladium species rather than metallic palladium. Electrochemical behavior depended strongly on composition. The hydroxyapatite coating containing 2 wt% palladium showed the lowest corrosion current density, 1.46 ± 0.02 µA cm^−2^ (0.0127 ± 0.0002 mm year^−1^), and a mean total resistance of 15.73 ± 0.13 kΩ cm^2^, close to that of the unmodified hydroxyapatite coating (1.95 ± 0.04 µA cm^−2^; 16.52 ± 0.40 kΩ cm^2^). The hydroxyapatite/graphene oxide coating showed the weakest barrier behavior, with a corrosion current density of 4.21 ± 0.07 µA cm^−2^ (0.0366 ± 0.0006 mm year^−1^) and a mean total resistance of only 8.50 ± 0.15 kΩ cm^2^. Microscopic and electrochemical evidence indicated that the expected barrier effect of graphene oxide was lost because the graphene oxide-containing layer was structurally heterogeneous. This layer contained folded, sheet-like domains and pathways that remained accessible to the electrolyte, and restacking or aggregation of the sheets induced by calcium ions is proposed as a plausible origin of this morphology. Adding 1 wt% palladium partially restored coating coverage and raised the mean total resistance to 14.97 ± 0.20 kΩ cm^2^ (2.15 ± 0.04 µA cm^−2^), whereas 2 wt% palladium lowered the interfacial charge-transfer resistance and reduced the mean total resistance to 11.73 ± 0.30 kΩ cm^2^ (2.91 ± 0.06 µA cm^−2^), even though the outer part of the coating remained intact. All coatings supported higher short-term A7R5 cell densities than the uncoated substrate, and the highest value, 330.2% of the control, was obtained for the graphene oxide coating containing 2 wt% palladium. The same graphene-oxide/palladium coatings, however, produced considerably stronger bioluminescent responses from *Staphylococcus aureus* than the remaining compositions. Overall, the hydroxyapatite coating containing 2 wt% palladium offered the most balanced combination of electrochemical and biological performance. The results show that the behavior of these coatings is governed by the organization of graphene oxide, by the interfacial chemistry associated with palladium, by the pathways available to the electrolyte, and by buried charge-transfer processes, rather than by nominal additive content alone. Their architecture is characterized by folded sheet-like domains and accessible electrolyte pathways, with Ca^2+^-mediated restacking/aggregation proposed as a plausible origin. Incorporation of 1 wt% Pd partially restored coating coverage and increased the mean total resistance to 14.97 ± 0.20 kΩ cm^2^, whereas increasing Pd to 2 wt% reduced the interfacial charge-transfer resistance and decreased the mean total resistance to 11.73 ± 0.30 kΩ cm^2^ despite retention of the outer-coating barrier. All coatings supported higher short-term A7R5 cell densities than the uncoated substrate, with HA/GO-2Pd reaching 330.2% of the control. However, the GO/Pd coatings produced substantially higher Xen36 bioluminescent responses than the remaining compositions. Overall, HA-2Pd provided the most balanced electrochemical and biological performance, while the results demonstrate that the behavior of HA/GO/Pd coatings is governed by GO organization, Pd-associated interfacial chemistry, electrolyte-access pathways, and buried interfacial charge-transfer processes rather than nominal additive content alone.

## 1. Introduction

Titanium and its alloys have long been established as metallic materials for load-bearing orthopedic and dental applications because of their favorable specific strength, corrosion resistance, and biological tolerance. Nevertheless, the comparatively high elastic modulus of conventional α and α + β titanium alloys remains an important limitation because the resulting mechanical mismatch with cortical bone may contribute to stress shielding and unfavorable long-term load transfer. These considerations have accelerated the development of metastable β-type titanium alloys, whose body-centered cubic structure generally permits a lower elastic modulus while retaining the strength and corrosion resistance required for load-bearing applications [1,2,3]. Among the alloying strategies developed for this purpose, Ti–Zr–Mo systems are of particular interest because Zr and Mo are considered comparatively suitable alloying elements for biomedical Ti alloys, while Mo acts as an effective β-stabilizer and Zr contributes to solid-solution strengthening and modification of the passive-film chemistry [1,2,3,4].

Recent investigations of Ti–Zr–Mo alloys have demonstrated that phase constitution, Zr/Mo content, and thermomechanical history strongly affect their mechanical and electrochemical behavior [4,5,6]. Within this alloy family, Ti–30Zr–5Mo has emerged as a promising biomedical composition in which the relative fractions of α and β phases can be controlled through processing and heat treatment. The β-dominant condition has been associated with improved electrochemical stability compared with more heterogeneous α-containing microstructures, emphasizing the importance of phase homogeneity for suppressing localized microgalvanic effects [5,6]. Surface-engineering studies on Ti–30Zr–5Mo have further demonstrated that bioactive coatings can substantially alter wettability, corrosion behavior, coating adhesion, and apatite-forming ability without changing the favorable bulk characteristics of the underlying alloy [7]. Thus, while β-Ti30Zr5Mo offers an attractive structural platform, its long-term biomedical performance can potentially be further improved by engineering the implant–tissue interface.

Hydroxyapatite (HA, Ca_10_(PO_4_)_6_(OH)_2_) remains one of the most extensively investigated ceramic materials for this purpose because of its chemical and crystallographic similarity to the inorganic mineral component of bone [8,9,10,11,12]. When applied as a surface coating, HA can provide an osteoconductive interface while simultaneously separating the metallic substrate from the surrounding physiological electrolyte. The effectiveness of this approach, however, depends strongly on coating crystallinity, phase purity, morphology, porosity, adhesion, and interfacial stability [8,9,10,11,12]. A wide range of coating techniques has therefore been investigated, including plasma spraying, sol–gel processing, electrophoretic deposition, electrochemical deposition, and hydrothermal synthesis [8,9,10,11,12,13,14,15,16,17,18,19]. Among these routes, hydrothermal processing is particularly attractive because crystalline calcium-phosphate structures can be generated under comparatively moderate processing conditions while limiting the thermal decomposition and residual-stress problems associated with high-temperature deposition [13,14,15]. Hydrothermal treatment also provides considerable control over HA nucleation and crystal growth and has been shown to enhance the bioactive surface characteristics of Ti-based substrates [13,14,15]. Electrochemical and related low-temperature deposition studies have similarly demonstrated that HA coatings can improve surface bioactivity and, when sufficiently continuous, contribute to the corrosion protection of biomedical Ti alloys [16,17,18,19].

Despite these advantages, pristine HA does not necessarily provide optimal mechanical, electrochemical, and biological functionality simultaneously. Local defects, coating porosity, brittleness, and insufficient antibacterial performance remain important challenges, motivating the incorporation of secondary phases into HA matrices [8,10,11]. Graphene oxide (GO) is particularly attractive in this context because its two-dimensional carbon framework contains abundant hydroxyl, epoxide, carbonyl, and carboxyl functionalities. These oxygen-containing groups can interact with calcium-containing species and provide sites for heterogeneous HA nucleation, while the sheet-like morphology can alter coating compactness, crack propagation, interfacial transport, and surface wettability [20,21,22,23]. GO/HA composite coatings produced on Ti-based substrates have consequently shown improvements in coating adhesion, mineralization behavior, mechanical integrity, cellular response, and, in several systems, corrosion resistance compared with pristine HA [20,21,22,23,24,25].

The influence of GO, however, is highly dependent on its concentration, dispersion state, deposition route, and the microstructure produced within the HA matrix. Electrophoretically deposited GO/HA coatings have shown reduced cracking and improved adhesion and electrochemical resistance [20], whereas electrochemically deposited GO/HA coatings have demonstrated increased HA crystallinity, bonding strength, and in vitro biological performance [21]. Graphene-containing HA coatings have also exhibited increased hardness, fracture resistance, and corrosion stability [22]. In hydrothermally synthesized HA/graphene systems, graphene-derived additions can modify HA nucleation and surface morphology and can improve corrosion behavior within an appropriate compositional range [23]. Nevertheless, the addition of a carbon nanostructure does not guarantee a monotonic improvement in coating properties. Excessive carbonaceous-phase incorporation, inadequate dispersion, local agglomeration, or disruption of continuous HA growth may generate heterogeneous electrolyte pathways and reduce the barrier effectiveness of the coating. The diverse responses reported for GO/HA, graphene/HA, and related carbon-reinforced calcium-phosphate coatings confirm that composition and processing must be considered together when interpreting corrosion and biological behavior [24,25,26,27,28,29,30,31,32,33].

In addition to carbon-based reinforcement, incorporation of metallic or ionic species into HA provides another strategy for controlling surface functionality. Noble-metal-containing calcium-phosphate systems are particularly interesting because the chemical state, dispersion, and interaction of the metal with the HA lattice or surface can substantially influence interfacial reactions. Palladium (Pd), although much less extensively investigated in biomedical HA coatings than Ag, Cu, or Zn, has been successfully associated with hydroxyapatite in several forms [34,35,36]. A ternary HA/rGO/Pd nanocomposite coating has previously been produced on stainless steel by electrodeposition, where incorporation of rGO and Pd was associated with improved corrosion resistance and cellular response relative to less complex coating compositions [37]. However, that study employed reduced graphene oxide, a stainless-steel substrate, and an electrodeposition route, and primarily focused on the resulting performance of the ternary coating. The behavior of Pd within a hydrothermally grown GO-containing HA matrix, particularly the relationship among GO organization, Pd-associated surface chemistry, coating continuity, and buried interfacial charge transfer, remains insufficiently understood.

Despite the increasing use of GO and metal-modified calcium-phosphate coatings, their combined effects cannot necessarily be predicted from the behavior of the individual constituents. The corrosion-protective function of GO depends strongly on nanosheet dispersion, orientation, and integration within the surrounding matrix. In Ca^2+^-rich environments, oxygen-containing groups on GO can participate in ion-mediated interactions that promote sheet association or restacking [38,39], potentially altering HA nucleation and producing heterogeneous transport pathways. The introduction of Pd adds a further level of complexity because its influence depends not only on concentration but also on its chemical state and its interaction with oxygen-containing GO environments [40]. Consequently, a GO/Pd-containing HA coating may exhibit substantially different interfacial electrochemistry from either HA/GO or HA/Pd alone. The relationship between GO organization, Pd chemical state, coating morphology, buried interfacial charge transfer, and biological response in hydrothermally deposited HA/GO/Pd coatings therefore remains insufficiently resolved.

In the present study, a systematic six-member coating series comprising HA, HA-1Pd, HA-2Pd, HA/GO, HA/GO-1Pd, and HA/GO-2Pd was hydrothermally deposited on β-Ti30Zr5Mo alloy. The study was designed not merely to identify the composition giving the highest performance, but to determine why GO and Pd produce composition-dependent and potentially non-additive responses within the HA matrix. X-ray diffraction (XRD), Fourier-transform infrared spectroscopy (FTIR), Raman spectroscopy, X-ray photoelectron spectroscopy (XPS), and scanning electron microscopy (SEM)–energy-dispersive X-ray spectroscopy (EDS) were combined to resolve phase constitution, GO retention, Pd chemical state, coating morphology, and elemental distribution. Electrochemical impedance spectroscopy (EIS) and potentiodynamic polarization (PDP) were then used to distinguish outer-coating barrier resistance from interfacial charge-transfer behavior, while post-polarization SEM–EDS was employed to assess the resulting morphological and compositional evolution. Short-term A7R5 cell-density measurements and *S. aureus* Xen36 bioluminescence imaging were further used to determine whether electrochemical optimization translated into corresponding biological performance. By integrating these datasets, the study establishes a mechanistic composition–structure–interface–property relationship for HA/GO/Pd coatings and evaluates the hypothesis that their multifunctional response is governed by the organization and chemistry of the GO–Pd–HA interface rather than by GO or Pd content alone.

## 2. Materials and Methods

### 2.1. Substrate Preparation

A metastable β-type Ti30Zr5Mo alloy was used as the substrate material. The alloy was sectioned into rectangular specimens with dimensions of approximately 10 × 20 × 3 mm. Prior to coating, the specimen surfaces were sequentially ground using 400-, 800-, 1200-, and 2000-grit SiC abrasive papers and subsequently polished with a 1 μm diamond suspension to obtain a uniform, near-mirror surface finish.

The mechanically prepared specimens were ultrasonically cleaned in acetone, ethanol, and deionized water for 10 min in each medium to remove residual contaminants and abrasive particles. The samples were then dried at room temperature.

Prior to hydrothermal coating, the substrates were chemically activated by immersion in 5 M NaOH solution at 60 °C for 12 h. Following activation, the specimens were thoroughly rinsed with deionized water and dried at 60 °C. This treatment was used to generate a hydroxyl-rich surface favorable for subsequent hydroxyapatite nucleation during hydrothermal processing.

### 2.2. Preparation of Ha, Ha-Pd, Ha/Go, and Ha/Go-Pd Precursor Suspensions

Calcium nitrate tetrahydrate (Ca(NO_3_)_2_·4H_2_O), diammonium hydrogen phosphate ((NH_4_)_2_HPO_4_), palladium(II) nitrate dihydrate (Pd(NO_3_)_2_·2H_2_O), and graphene oxide (GO) powder were obtained from Sigma-Aldrich and used as received. For each coating batch, 1.417 g of Ca(NO_3_)_2_·4H_2_O was dissolved in 30 mL of deionized water, corresponding to a 0.20 M Ca precursor solution. The phosphate precursor amount was fixed at 0.475 g of (NH_4_)_2_HPO_4_, providing a Ca/P molar ratio of approximately 1.67.

For the GO-free groups (HA, HA-1Pd, and HA-2Pd), 0.475 g of (NH_4_)_2_HPO_4_ was dissolved in 30 mL of deionized water, corresponding to a phosphate precursor concentration of 0.12 M. The separately prepared calcium and phosphate solutions were subsequently combined, giving a total precursor volume of 60 mL.

For the GO-containing groups (HA/GO, HA/GO-1Pd, and HA/GO-2Pd), the same phosphate precursor amount (0.475 g) was dissolved in 20 mL of deionized water. Separately, the required amount of solid GO powder was dispersed in 10 mL of deionized water and ultrasonicated for 1 h to promote homogeneous dispersion and minimize sheet agglomeration. The phosphate solution and GO dispersion were then combined with the 30 mL calcium precursor solution. Thus, the total precursor volume was maintained at 60 mL for all coating compositions, while the total Ca and P amounts and the nominal Ca/P molar ratio were kept constant.

The GO loading was defined on a mass basis relative to the combined calculated elemental masses of Ca and P and was maintained at a nominal value of 1.0 wt% in all GO-containing formulations. The GO content therefore refers to the nominal amount introduced into the precursor suspension rather than to the final GO concentration of the deposited coating.

Palladium was introduced using solid Pd(NO_3_)_2_·2H_2_O as the precursor. The nominal Pd loadings of 1 and 2 wt% refer to elemental Pd relative to the same combined elemental Ca + P mass basis. The required Pd(NO_3_)_2_·2H_2_O amount was calculated from its elemental Pd fraction and was added after preparation and combination of the other precursor components under continuous magnetic stirring.

The pH of the resulting precursor suspensions was adjusted to approximately 10–11 by gradual addition of aqueous ammonia solution (Sigma-Aldrich) under continuous stirring. Six coating compositions were subsequently obtained: HA, HA-1Pd, HA-2Pd, HA/GO, HA/GO-1Pd, and HA/GO-2Pd.

The nominal compositions are summarized in Table 1.

### 2.3. Hydrothermal Coating Process

The prepared precursor suspensions were transferred into Teflon-lined stainless-steel autoclaves (Fytronix, Elazığ, Türkiye). The chemically activated Ti30Zr5Mo alloy substrates were positioned vertically within the precursor solutions to allow uniform exposure of the substrate surfaces during hydrothermal treatment.

The sealed autoclaves were maintained at 180 °C for 24 h. Under hydrothermal conditions, calcium- and phosphate-containing species nucleated and crystallized on the activated substrate surface, resulting in the formation of HA-based coatings with or without GO and Pd incorporation.

After completion of the hydrothermal process, the autoclaves were allowed to cool naturally to room temperature. The coated specimens were then removed, gently rinsed with deionized water to eliminate loosely attached particles, and dried in an oven at 80 °C prior to characterization.

### 2.4. Structural, Chemical, and Morphological Characterization

The phase composition of the coatings was investigated by grazing-incidence X-ray diffraction (GIXRD) using a Rigaku SmartLab diffractometer with Cu-Kα radiation (λ = 1.5406 Å), operated at 40 kV and 40 mA. Diffraction patterns were collected at an incidence angle of 0.5° over a 2θ range of 5–80° using a step size of 0.01°. The complete measured range was examined for every specimen, and no reflection was detected between 5° and 10° or between 70° and 80° in any of the six coatings. Figure 1 therefore reproduces the diagnostically informative 10–70° interval, which contains every reflection recorded in this work. The patterns are shown as recorded, without background subtraction or intensity normalization, and are vertically offset for clarity; the offset is applied for display purposes only and carries no quantitative meaning. The gradual decay of the background towards low angles is the air- and holder-scattering contribution characteristic of the fixed low incidence angle used in grazing-incidence geometry.

Chemical bonding and functional groups were examined by Fourier-transform infrared spectroscopy using a Thermo Scientific Nicolet iS5 spectrometer (Thermo Fisher Scientific, Madison, WI, USA) in attenuated total reflection (ATR) mode. Spectra were recorded over 4000–500 cm^−1^ at a spectral resolution of 4 cm^−1^ using 32 scans per sample.

Raman spectroscopy was performed using a HORIBA Jobin Yvon LabRAM ARAMIS system (HORIBA Jobin Yvon S.A.S., Villeneuve d’Ascq, France) with a 532 nm excitation laser. Spectra were collected over the 400–2000 cm^−1^ range to identify phosphate-related vibrational modes and the characteristic carbon D and G bands associated with GO-containing coatings.

Surface chemical composition and chemical states were investigated by X-ray photoelectron spectroscopy (XPS) using a PerkinElmer PHI 5600 ESCA system (PerkinElmer Inc., Waltham, MA, USA) equipped with an Al Kα X-ray source. Detailed XPS analysis was performed on the HA/GO-2Pd coating, selected as the GO-containing composition with the highest nominal Pd loading, to evaluate the surface chemical states of the graphene-derived carbon phase and incorporated Pd. Survey spectra were acquired using a pass energy of 160 eV to identify the principal surface elements, followed by high-resolution scans of the Ca 2p, P 2p, C 1s, O 1s, and Pd 3d regions using a pass energy of 20 eV and an energy step size of 0.05 eV. The binding-energy scale was referenced to the C 1s C–C/C=C component at 284.4 eV. Spectral processing and peak deconvolution were performed using CasaXPS software (version 2.3.27PR5.4; Casa Software Ltd., Teignmouth, UK). A Tougaard background was applied, and the high-resolution components were fitted using Gaussian–Lorentzian line shapes.

Surface morphology and elemental distribution were examined by field-emission scanning electron microscopy using a JEOL JSM-7001F microscope (JEOL Ltd., Akishima, Tokyo, Japan) equipped with energy-dispersive X-ray spectroscopy. SEM observations and EDS analyses were performed at an accelerating voltage of 15 kV. Elemental point analyses and mapping were conducted on the as-deposited coatings and on specimens retrieved after potentiodynamic polarization testing.

### 2.5. Electrochemical Measurements

Electrochemical measurements were performed using a Gamry PCI14/750 potentiostat/galvanostat (Gamry Instruments, Warminster, PA, USA) in a conventional three-electrode electrochemical cell. The coated specimen served as the working electrode, with an exposed surface area of approximately 0.28 cm^2^. A platinum wire and a saturated Ag/AgCl electrode were used as the counter and reference electrodes, respectively.

Hank’s balanced salt solution maintained at 37 ± 0.5 °C and approximately pH 7.4 was used as the electrolyte to simulate physiological conditions. Prior to electrochemical measurements, the open-circuit potential (OCP) was monitored for approximately 1800 s until a near-steady-state potential was established.

Electrochemical impedance spectroscopy was subsequently performed at OCP over a frequency range of 100 kHz to 0.01 Hz using a sinusoidal perturbation amplitude of 10 mV. The experimental EIS data were fitted using Echem Analyst software (version 7.11; Gamry Instruments, Warminster, PA, USA).

The spectra were interpreted using the two-time-constant equivalent electrical circuit:

Rs(Qc(Rcoat(QdlRct)))

where *Rs* represents the solution resistance, *Qc* is the constant-phase element associated with the coating layer, *Rcoat* represents the coating resistance, *Qdl* represents the non-ideal double-layer capacitive response, and *Rct* is the charge-transfer resistance associated with electrochemically active regions at or near the coating/substrate interface.

Potentiodynamic polarization measurements were initiated at −0.30 V relative to the stabilized open-circuit potential and scanned in the anodic direction at a rate of 1 mV s^−1^. The anodic scans extended to approximately 2.7–2.9 V vs. Ag/AgCl depending on the specimen response.

The corrosion potential (*Ecorr*), corrosion current density (*icorr*), anodic and cathodic Tafel slopes (βa and βc), polarization resistance (*Rp*), and corrosion rate were determined by Tafel extrapolation using Echem Analyst in accordance with ASTM G102-23 [23].

The anodic and cathodic Tafel regions were selected within potential intervals of approximately 50–150 mV on either side of the corrosion potential, in segments that remained linear on a semi-logarithmic current scale over at least one decade of current density and that were free of the curvature associated with the onset of passivation or with mass-transport control. The same selection criteria were applied to every specimen so that the comparison between coating compositions remained internally consistent. Corrosion rates were calculated in accordance with ASTM G102 using the constant 3.27 × 10^−3^ mm g µA^−1^ cm^−1^ year^−1^ together with an equivalent weight of 11.97 g eq^−1^ and a density of 4.51 g cm^−3^, corresponding to the titanium-rich substrate oxidized in the tetravalent state. If the equivalent weight and density of the alloy are instead calculated from its nominal composition according to ASTM G102 (14.49 g eq^−1^ and 5.13 g cm^−3^), every corrosion rate is scaled by a single constant factor of 1.06, so that the ranking of the coatings and all of the conclusions drawn from it remain unchanged. Because the coatings are not fully dense and the polarized area contains both coated and locally accessible substrate regions, the corrosion rates reported here are treated throughout as comparative indicators rather than as absolute metal-loss rates.

Because the polarization curves did not exhibit a sufficiently extended and stable passive-current plateau followed by a clearly defined breakdown event, passive current density and pitting potential were not reported.

Following potentiodynamic polarization testing, selected specimens were re-examined by SEM and EDS to evaluate morphological changes and the retention of coating-associated elements after electrochemical exposure.

Electrochemical measurements were performed using three independently prepared specimens for each coating composition (n = 3). Potentiodynamic polarization parameters are reported as mean ± standard deviation (SD). For the impedance measurements, the total resistance was evaluated for each of the three specimens and is reported as mean ± SD; this quantity provides the statistical basis for the comparison between compositions. The individual equivalent-circuit parameters listed are taken from a representative fit, namely that of the specimen whose total resistance lay closest to the mean of its group. This convention was adopted because the resistive and constant-phase-element parameters obtained within a single fit are mutually correlated, so that averaging them across separate fits can produce a parameter set that does not itself reproduce any measured spectrum. The standard deviations of the total resistance, which lie between approximately 1 and 3% of the corresponding mean values, confirm that the three specimens of each composition behaved consistently and that the representative fit is not an outlier.

### 2.6. In Vitro Cell Response

The in vitro cellular response of the uncoated and coated specimens was evaluated using A7R5 rat aortic smooth-muscle cells (ATCC CRL-1444). The A7R5 assay was employed as a comparative mammalian cell–surface response model to assess short-term cell attachment and surface-associated cell density; it was not intended to evaluate osteogenic differentiation or bone-forming activity.

Before the cellular and bacterial experiments, all uncoated and coated specimens were sterilized under a laminar-flow cabinet by ultraviolet irradiation for 30 min on each face. A dry, non-thermal route was selected deliberately in preference to autoclaving, because saturated steam at 121 °C can hydrate the apatite surface and alter its morphology, and in preference to prolonged immersion in ethanol, because a liquid step can leach weakly bound species from a porous coating. Ultraviolet irradiation at the doses used for surface sterilization does not supply sufficient energy to reduce graphene oxide, to alter the oxidation state of palladium, or to modify the apatite morphology, and the surfaces presented to the cells and the bacteria are therefore taken to be those characterized in Section 3.1, Section 3.2, Section 3.3, Section 3.4 and Section 3.5. No independent post-sterilization characterization was carried out, and this is stated among the limitations in Section 3.10.

Cells were maintained in Dulbecco’s Modified Eagle’s Medium (DMEM; Gibco, Thermo Fisher Scientific, Waltham, MA, USA) supplemented with 10% fetal bovine serum, 2 mM L-glutamine, and 1% penicillin–streptomycin under humidified conditions at 37 °C and 5% CO_2_.

For the surface-response experiments, cells were seeded onto the uncoated and coated specimens at an initial density of approximately 1 × 10^4^ cells cm^−2^ and cultured for 24 h.

Following incubation, cell attachment, spreading, morphology, and surface coverage were evaluated by phase-contrast microscopy. Quantitative analysis was performed by counting the cells within calibrated microscopic fields and normalizing the counts to the corresponding imaged area. The results were expressed as estimated cell density (cells cm^−2^).

To facilitate comparison among the experimental groups, the cellular response was additionally normalized to that of the uncoated Ti30Zr5Mo substrate, which was defined as 100%. Identical imaging conditions, magnification, field area, and counting procedures were applied to all groups.

Because the quantitative values were derived from microscopic cell counts rather than a metabolic viability assay, the normalized percentages are interpreted herein as relative cell-density/cellular-response values rather than direct metabolic viability measurements.

Cellular-response experiments were performed using three independently prepared specimens for each experimental group (n = 3). Quantitative cell-density results are reported as mean ± standard deviation (SD), and relative cell-density values were calculated by normalizing the corresponding mean values to the uncoated β-Ti30Zr5Mo control.

### 2.7. Bacterial Bioluminescence Response Evaluation

The bacterial response to the coating surfaces was evaluated using the bioluminescent *Staphylococcus aureus* Xen36 strain. Prior to the surface-inoculation experiments, the bacterial culture was prepared under sterile conditions in Brain Heart Infusion (BHI) medium supplemented with kanamycin (200 µg mL^−1^) to maintain selective pressure for the bioluminescent phenotype. Kanamycin was used only during this pre-culture stage and was not included during the subsequent incubation of the bacteria with the coated specimens.

Following pre-culture, the bacterial suspension was adjusted to approximately 1 × 10^4^ CFU mL^−1^. The coated specimens were inoculated with the prepared bacterial suspension and incubated at 37 °C for 24 h to allow bacterial attachment and surface-associated growth.

After the 24 h incubation and before imaging, each specimen was rinsed gently in sterile phosphate-buffered saline to remove planktonic and loosely attached bacteria and was then transferred to a clean, sterile surface for acquisition so that no incubation medium lay within the region of interest. The recorded photon flux is therefore attributed to bacteria associated with the specimen surfaces. The experiment did not include the uncoated alloy or a positive antibacterial control, so the bioluminescent responses are comparable within the coating series but cannot be referred to an absolute scale; this is stated among the limitations in Section 3.10.

After incubation, bacterial bioluminescence was recorded using an in vivo imaging system (IVIS). An identical region of interest was applied to each specimen, and the emitted signal was quantified as total radiant flux (photons s^−1^).

Because the Xen36 lux system generates bioluminescence from viable, metabolically active bacteria, the measured photon flux was used as a comparative indicator of bacterial activity on the different coating surfaces. Accordingly, the IVIS results were interpreted as relative bacterial bioluminescence responses rather than as direct colony-forming-unit counts.

Bacterial bioluminescence experiments were performed using three independently prepared specimens for each coating composition (n = 3), with IVIS measurements conducted under identical acquisition and region-of-interest conditions.

All coating compositions were independently prepared in triplicate (n = 3). Electrochemical, cellular, and bacterial-response analyses were performed using three independently prepared specimens for each experimental group. The independently prepared specimen was considered the experimental unit, and quantitative results are reported as mean ± standard deviation (SD) across the three independent specimens. Where statistical comparisons among groups were performed, one-way analysis of variance (ANOVA) followed by Tukey’s post hoc test was used, with differences considered statistically significant at *p* < 0.05.

## 3. Results and Discussion

### 3.1. Phase Composition and Structural Evolution

The XRD patterns of the HA, HA-1Pd, HA-2Pd, HA/GO, HA/GO-1Pd, and HA/GO-2Pd coatings are presented in Figure 1.

All coating compositions exhibited the characteristic diffraction profile of crystalline hydroxyapatite, demonstrating that neither Pd addition nor GO incorporation suppressed the formation of the apatite phase under the applied hydrothermal conditions. The principal reflections were indexed to the (002), (102), (210), (211), (300), (202), (301), (222), (312), (213), and (004) planes of HA, with the intense group of reflections in the 31–33° region and particularly the (211) plane representing the dominant apatite contribution. Comparable HA phase development has been reported for GO/HA coatings produced on Ti-based substrates using electrochemical, electrophoretic, and hydrothermal routes [20,21,22,23].

No pronounced emergence of secondary calcium-phosphate phases was observed as a systematic consequence of either GO or Pd incorporation. This finding indicates that the hydrothermal conditions used in the present study were sufficiently favorable for apatite crystallization throughout the entire compositional series. More importantly, preservation of the principal HA diffraction pattern after modification suggests that the relatively low GO content and the 1–2 wt% Pd additions altered the local coating chemistry without causing extensive transformation of the HA matrix into a different crystalline calcium-phosphate structure. Similar retention of the HA crystal structure following GO incorporation has been reported for GO-reinforced HA coatings, although changes in peak width, relative intensity, and crystallinity can occur depending on GO concentration and deposition conditions [20,21,26].

A distinct low-angle reflection in the approximately 10–12° region was observed in the GO-containing coatings and was absent from the GO-free HA, HA-1Pd, and HA-2Pd groups. This feature is consistent with the expanded interlayer spacing generated by oxygen-containing functional groups within stacked graphene oxide sheets and therefore provides structural evidence for the retention of GO after hydrothermal processing. A weaker and broader contribution was also present in the approximately 21–23° region. This region contains the (200) and (111) reflections of hydroxyapatite, so any carbon-related contribution is superimposed on apatite reflections and cannot be separated from them; both contributions are indicated in Figure 1 and listed together in Table 2. Because diffraction features in this region may vary considerably with the oxidation state, stacking order, partial reduction, and interaction of GO sheets with the inorganic HA matrix, this contribution is considered supportive rather than independently diagnostic of GO. The presence of the carbonaceous phase is therefore evaluated more conclusively using the D and G bands observed by Raman spectroscopy in Section 3.3.

Weak diffraction contributions were additionally observed close to approximately 40° and 46–47° in the Pd-containing coatings, and these positions are marked in Figure 1 as overlapping contributions. They correspond to the characteristic (111) and (200) reflections reported for face-centered-cubic Pd-containing systems [34]. However, these regions also overlap strongly with reflections originating from HA and the β-Ti30Zr5Mo substrate. Consequently, the XRD patterns alone do not provide sufficient chemical-state specificity to determine whether Pd is present as metallic Pd, an oxidized Pd-containing phase, or highly dispersed/lattice-associated species. The chemical state of palladium is therefore interpreted primarily from the high-resolution Pd 3d XPS spectra discussed in Section 3.4.

Every reflection detected over the measured 5–80° range has now been assigned; the assignments are collected in Table 2. Between approximately 42° and 48°, the observed reflections correspond to the (311), (113), (203), (222), and (312) planes of hydroxyapatite (ICDD card 09-0432); in the Pd-containing coatings, the (200) reflection of face-centered-cubic palladium, expected at approximately 46.7°, overlaps with the hydroxyapatite (222) reflection in the same window. Between approximately 52° and 55°, the (402) and (004) reflections of hydroxyapatite are superimposed on the low-angle flank of the β-(200) reflection of the Ti–30Zr–5Mo substrate, which is located at approximately 55.5° for the lattice parameter of the present alloy. Between 60° and 70°, the only feature rising clearly above the background is the β-(211) reflection of the substrate at approximately 69.7°, with which the (220) reflection of palladium expected near 68° overlaps; the weak hydroxyapatite reflections of the (511)/(304) group expected in the same interval remain below the detection limit of the present patterns. The reflection rising at the right-hand edge of Figure 1 is the β-(211) reflection of the substrate, centered at approximately 69.7°, and it is labeled as such. The patterns were acquired to 80°, and the interval between 70° and 80° contains no reflection in any of the six coatings, just as the interval between 5° and 10° contains none; the displayed range therefore omits no phase, and the apparent prominence of this reflection at the edge of the figure reflects only the position of the display limit.

These medium- and high-angle reflections were systematically more pronounced for the HA/GO coating than for the remaining compositions, and two complementary factors account for this observation. First, the reflections located at approximately 55.5° and 69–70° originate from the β-Ti30Zr5Mo substrate, and their increased intensity for HA/GO is consistent with the reduced coating continuity of this composition. The same conclusion follows independently from the pronounced substrate-derived Ti and Mo signals recorded by energy-dispersive X-ray spectroscopy for HA/GO (Section 3.5) and from the lowest total impedance of the series measured for the same coating (Section 3.6.1). Second, the weaker apatite reflections between approximately 42° and 55° gained intensity relative to the dominant (211) reflection in the GO-containing coating, which indicates a reduction in the c-axis preferred orientation that characterizes the GO-free coatings. Oxygen-containing functional groups on the graphene oxide sheets chelate Ca^2+^ and create a high density of nucleation sites distributed on and between the sheets, so that apatite crystallites nucleate in comparatively random orientations instead of growing as textured, c-axis-aligned crystals directly on the metal surface. A comparable loss of texture, accompanied by an increase in the relative intensity of the secondary reflections, has been reported for graphene-oxide-reinforced apatite coatings [20,21,26]. Both effects are therefore consistent with the heterogeneous and incompletely covering HA/GO architecture that governs the electrochemical behavior described in Section 3.6, and neither of them requires the formation of an additional crystalline phase.

Reflections attributable to the Ti30Zr5Mo substrate were detected in all six diffraction patterns, particularly in the regions associated with the β-Ti (110), (200), and (211) planes. The presence of these substrate reflections is consistent with the metastable β-type structure reported for appropriately solution-treated Ti–30Zr–5Mo alloys [5,6]. Their detection despite the use of grazing-incidence XRD indicates that the hydrothermally produced coatings remained sufficiently thin and/or locally X-ray transparent for a contribution from the underlying alloy to be recorded. Similar substrate contributions have been reported for comparatively thin or locally porous HA/GO coatings on Ti substrates [21,26].

Among the present coatings, the substrate-related contribution was comparatively pronounced for the HA/GO composition. This observation is consistent with the elevated Ti- and Mo-related signals subsequently detected by EDS for this coating and may indicate a comparatively less continuous or locally more permeable HA/GO architecture. Nevertheless, because the diffraction profiles were vertically offset for visual comparison and no independent coating-thickness measurement was available, differences in substrate-peak intensity should not be interpreted quantitatively as direct differences in coating thickness. Instead, the XRD and EDS observations together suggest differences in coating coverage or microstructural continuity that are further evaluated by SEM and electrochemical measurements.

Overall, the XRD results demonstrate that hydrothermal processing produced an HA-dominated crystalline coating in every composition while retaining identifiable signatures associated with GO and the β-Ti30Zr5Mo substrate. The absence of a major phase transformation upon Pd addition is particularly relevant to the subsequent electrochemical analysis: the substantial differences in corrosion behavior among HA, Pd-modified HA, and GO/Pd-modified HA cannot be explained simply by the formation or disappearance of the principal HA phase. Rather, they are more likely associated with changes in surface chemical state, coating morphology, local continuity, and interfacial electrochemical heterogeneity, which are examined using Raman spectroscopy, XPS, SEM–EDS, EIS, and potentiodynamic polarization in the following sections.

### 3.2. Chemical Bonding and Functional-Group Characteristics

The FTIR spectra of the HA, HA-1Pd, HA-2Pd, HA/GO, HA/GO-1Pd, and HA/GO-2Pd coatings are presented in Figure 2, while the corresponding vibrational assignments are summarized in Table 3. All coating compositions exhibited the characteristic vibrational features of hydroxyapatite, confirming preservation of the apatite chemical framework after incorporation of GO and Pd.

The most intense absorption envelope was observed in the approximately 1020–1092 cm^−1^ region and was assigned to the ν_3_ asymmetric stretching vibration of PO_4_^3−^. A shoulder at approximately 962–963 cm^−1^ corresponded to the ν_1_ symmetric stretching mode of phosphate. At lower wavenumbers, the bands located at approximately 559–565 and 600–630 cm^−1^ were attributed to the ν_4_ bending vibrations of PO_4_^3−^. The persistence of these characteristic phosphate vibrations in all six coatings agrees with the XRD results and confirms that neither GO incorporation nor the addition of 1–2 wt% Pd caused extensive disruption of the HA framework.

A broad absorption band extending over approximately 3200–3400 cm^−1^ was observed in all specimens and was primarily associated with O–H stretching from structural hydroxyl groups and adsorbed water. In the GO-containing coatings, hydroxyl-containing surface functionalities of GO may additionally contribute to this broad region. Because hydroxyl groups are present in both the HA matrix and GO, however, the O–H envelope alone cannot be used as an independent indicator of GO incorporation.

A contribution in the approximately 1580–1650 cm^−1^ region was particularly evident in the GO-containing coatings. In addition to the H–O–H bending vibration of adsorbed water expected near this region, the enhanced feature observed for HA/GO, HA/GO-1Pd, and HA/GO-2Pd is attributable to the skeletal C=C stretching vibration associated with sp^2^-hybridized carbon domains. The considerably weaker contribution in the GO-free HA, HA-1Pd, and HA-2Pd spectra supports the assignment of this enhanced band to the carbonaceous component of the composite coatings. Because the C=C vibration and the H–O–H bending mode occur within a partially overlapping spectral range, the feature should be regarded as a combined contribution rather than as a completely isolated GO peak. Nevertheless, its preferential enhancement in the GO-containing specimens provides clear supporting evidence for retention of the GO-derived carbon framework after hydrothermal processing.

Other oxygen-containing functionalities characteristic of GO are more strongly affected by spectral overlap with the HA matrix. C–O and epoxide-related vibrations expected in approximately the 1225–1266 cm^−1^ region occur on the shoulder of the intense phosphate absorption envelope and therefore cannot be reliably separated as independent peaks. Similarly, C–O/alkoxy-related contributions close to approximately 1038–1054 cm^−1^ overlap directly with the dominant ν_3_(PO_4_^3−^) vibration. Consequently, the absence of individually resolved C–O-related bands does not indicate loss of GO from the coatings. Instead, FTIR provides complementary chemical evidence that is strengthened by the characteristic D and G bands in the Raman spectra and by the oxygenated carbon components subsequently identified in the C 1s XPS spectra.

A group of weak features was also detected between approximately 2000 and 2400 cm^−1^ in all six spectra, and these are addressed here so that every feature visible in Figure 2 is accounted for. The sharp doublet centered at approximately 2340–2360 cm^−1^ corresponds to the asymmetric stretching vibration of gaseous carbon dioxide and originates from incomplete compensation of the atmospheric contribution in the attenuated-total-reflection sample compartment. Its intensity varied randomly between successive measurements and showed no relationship with either graphene oxide or palladium content, which confirms its instrumental rather than chemical origin. The considerably weaker and broader absorptions located between approximately 2000 and 2200 cm^−1^ are assigned to combination and overtone modes of the phosphate group, which are routinely observed as low-intensity features in this otherwise transparent region of apatite spectra. The weak bands detected between approximately 2850 and 2960 cm^−1^ are assigned to C–H stretching vibrations of residual organic species and of aliphatic groups associated with the graphene-oxide surface. None of these contributions reflects a chemical modification introduced by graphene oxide or palladium, and they are therefore excluded from the interpretation of the coating chemistry; they are nevertheless labeled in Figure 2 and listed in Table 3 for completeness.

A carbonate-related absorption was observed within approximately 1337–1400 cm^−1^ for the coating series. Interestingly, the position of this band varied with composition. In most Pd-containing coatings, the feature occurred at approximately 1337–1339 cm^−1^, whereas the HA/GO coating exhibited a shift toward approximately 1399 cm^−1^. This compositional dependence suggests that GO incorporation modifies the local bonding environment of carbonate-containing species within the hydrothermally formed HA matrix. However, because no corresponding transformation of the dominant HA phase was detected by XRD, this shift is more appropriately attributed to changes in local chemical coordination or surface environment than to formation of a distinct secondary calcium-phosphate phase.

The FTIR spectra also provide useful information regarding the influence of Pd on the HA framework. Increasing Pd content from 0 to 2 wt% did not result in disappearance of the characteristic phosphate vibrations or in major systematic shifts of the principal PO_4_^3−^ bands. This indicates that Pd incorporation did not cause large-scale destruction of the apatite structure detectable by FTIR. The substantial composition-dependent differences observed later in the electrochemical measurements must therefore arise predominantly from more localized changes in surface chemistry, coating morphology, interfacial heterogeneity, and charge-transfer behavior rather than from wholesale decomposition of the HA phase.

The weak band observed at approximately 2340–2360 cm^−1^ was attributed to atmospheric CO_2_ interference and was not considered representative of the coating chemistry.

Overall, the FTIR results confirm preservation of the HA phosphate framework across the entire coating series and provide additional evidence for GO incorporation through the enhanced C=C-related contribution observed in the GO-containing compositions. The partial overlap of GO-derived oxygen-containing vibrations with the strongly IR-active HA matrix highlights the importance of combining FTIR with Raman spectroscopy and XPS for an unambiguous interpretation of the carbonaceous phase and its chemical state.

### 3.3. Raman Spectra

The Raman spectra of the HA, HA-1Pd, HA-2Pd, HA/GO, HA/GO-1Pd, and HA/GO-2Pd coatings are presented in Figure 3. All coating compositions exhibited the characteristic Raman response of hydroxyapatite, with the most intense band located at approximately 960 cm^−1^ and assigned to the ν_1_ symmetric stretching vibration of PO_4_^3−^. Additional weaker phosphate-related bands corresponding to the ν_2_, ν_3_, and ν_4_ vibrational modes were also detected, confirming preservation of the apatite framework throughout the entire coating series.

The persistence of the intense ν_1_(PO_4_^3−^) band after incorporation of both GO and Pd is consistent with the XRD and FTIR results and indicates that neither modification caused substantial structural degradation of the HA phase. In particular, no systematic disappearance of the principal phosphate Raman bands was observed with increasing Pd content from 0 to 2 wt%, further supporting the conclusion that the changes in electrochemical behavior among the coating compositions cannot be attributed simply to loss of the HA structure.

A clear distinction between the GO-free and GO-containing coatings was observed in the carbon-related spectral region. HA/GO, HA/GO-1Pd, and HA/GO-2Pd exhibited two broad bands centered at approximately 1350 and 1590 cm^−1^, corresponding to the characteristic D and G bands of graphene-derived carbon structures. The D band is associated primarily with defect-activated breathing modes of sp^2^ carbon rings and therefore reflects structural disorder, edge sites, and disruption of the extended graphitic lattice. In contrast, the G band originates from the in-plane stretching vibration of sp^2^-bonded carbon atoms and represents the graphitic carbon framework.

The simultaneous presence of the D and G bands provides direct spectroscopic evidence that the graphene-derived carbon phase was retained within the HA matrix after hydrothermal processing. This observation is particularly important because several oxygen-containing GO vibrations partially overlap with the intense HA phosphate bands in the FTIR spectra. Raman spectroscopy therefore provides a more selective confirmation of the carbonaceous component and supports the assignment of the enhanced C=C-related FTIR contribution observed in the GO-containing coatings.

The broad nature of the D and G bands indicates that the carbonaceous phase retained a substantial degree of structural disorder, as expected for graphene oxide rather than highly ordered graphite. Hydrothermal treatment can modify the oxygen-containing functional groups and local sp^2^/sp^3^ carbon balance of GO; however, the present Raman spectra demonstrate that the graphitic carbon backbone remained detectable after coating synthesis. Because quantitative D- and G-band intensity ratios were not determined from the present dataset, no numerical (I_D/I_G) comparison among HA/GO, HA/GO-1Pd, and HA/GO-2Pd is made here. Accordingly, the spectra are interpreted primarily as qualitative evidence of GO retention rather than as a quantitative measure of defect density or reduction degree.

The addition of Pd did not eliminate the D and G bands in the GO-containing coatings, indicating that incorporation of 1 and 2 wt% Pd did not remove or completely disrupt the GO-derived carbon framework. Nevertheless, the preservation of the characteristic GO Raman signature does not necessarily imply identical spatial distribution or interfacial organization of GO in the three GO-containing coatings. Differences in GO sheet arrangement, interaction with Pd-containing species, and HA nucleation around the carbon phase may occur without producing major changes in the positions of the D and G bands. These microstructural effects are therefore examined more directly by SEM–EDS and subsequently correlated with the electrochemical response.

Overall, the Raman results confirm the coexistence of a crystalline HA matrix and a structurally disordered GO-derived carbon phase in the GO-containing coatings. Together with XRD, FTIR, and XPS, these findings demonstrate that the major compositional differences among the coatings arise from incorporation and local organization of GO and Pd rather than from replacement of the HA matrix by secondary phases.

### 3.4. Surface Chemical Composition and Chemical States

X-ray photoelectron spectroscopy was employed to clarify the surface chemical environment of the HA/GO-2Pd coating and, in particular, to determine the chemical states of the GO-derived carbon species and incorporated Pd. The survey spectrum shown in Figure 4A reveals the presence of Ca, P, O, C, and Pd, with approximate surface concentrations of 8.7 at% Ca, 6.1 at% P, 58.0 at% O, 21.0 at% C, and 2.7 at% Pd. The simultaneous detection of the characteristic HA elements together with a pronounced carbon contribution and detectable Pd confirms that both GO- and Pd-derived species were present within the surface region of the HA/GO-2Pd coating probed by XPS.

The comparatively high carbon concentration is particularly noteworthy. Although adventitious carbon inevitably contributes to the C 1s signal of air-exposed surfaces, the magnitude of the carbon signal together with its high-resolution chemical-state distribution indicates that the detected carbon cannot be attributed solely to surface contamination. Instead, the C 1s spectrum provides direct evidence for the GO-derived carbonaceous component within the composite coating.

The Ca 2p spectrum (Figure 4B) exhibited a characteristic spin–orbit doublet, with Ca 2p_3_/_2_ and Ca 2p_1_/_2_ components located at approximately 345.2 and 350.1 eV, respectively. The P 2p region (Figure 4C) was dominated by a component at approximately 132.9 eV associated with phosphate species, accompanied by a weaker contribution at approximately 134.1 eV. Together with the phosphate vibrational modes observed by FTIR and Raman spectroscopy and the HA reflections detected by XRD, these Ca and P signals confirm that the surface retained an apatite-dominated calcium-phosphate chemical environment.

More detailed information on the carbonaceous phase was obtained from the high-resolution C 1s spectrum (Figure 4D). The envelope was resolved into four contributions centered at approximately 284.4, 286.1, 287.6, and 289.1 eV. The dominant low-binding-energy component at approximately 284.4 eV was assigned to C–C/C=C bonding within sp^2^-rich carbon domains. The higher-binding-energy components at approximately 286.1 and 287.6 eV were attributed to C–O and C=O environments, respectively, while the component at approximately 289.1 eV was associated with O–C=O functionalities. The coexistence of sp^2^ carbon and multiple oxygen-containing carbon environments is characteristic of an oxidized graphene-derived structure and provides strong chemical evidence for retention of GO within the hydrothermally deposited HA matrix.

These results complement the FTIR and Raman analyses. FTIR revealed an enhanced C=C-related contribution in the GO-containing coatings, although several oxygenated carbon vibrations overlapped with the intense phosphate bands of HA. In contrast, XPS resolves C–O, C=O, and O–C=O environments individually, while Raman spectroscopy independently confirms the graphitic framework through the characteristic D and G bands. The combined use of these techniques therefore establishes GO incorporation more convincingly than any individual characterization method alone.

The O 1s spectrum (Figure 4E) further reflects the chemically heterogeneous oxygen environment of the composite coating. Deconvolution revealed components centered at approximately 531.8, 532.7, and 534.4 eV. The dominant contribution at approximately 531.8 eV was assigned mainly to oxygen associated with the phosphate-containing HA framework, whereas the component at approximately 532.7 eV was attributed to C–O/C–OH and related hydroxylated surface environments. The higher-binding-energy contribution at approximately 534.4 eV was associated predominantly with molecularly adsorbed water and weakly bound surface oxygen species. The presence of both phosphate- and carbon-associated oxygen environments is consistent with intimate chemical contact between the HA matrix and the oxygen-functionalized GO phase.

The most significant chemical-state information was obtained from the Pd 3d spectrum (Figure 4F). A well-resolved spin–orbit doublet was observed at approximately 338.0 eV for Pd 3d_5_/_2_ and 343.3 eV for Pd 3d_3_/_2_, corresponding to a spin–orbit separation of approximately 5.3 eV. Importantly, the Pd 3d_5_/_2_ binding energy is substantially higher than the approximately 335 eV region generally associated with metallic Pd^0^. Oxidized Pd species, including Pd^2+^/PdO-type environments and strongly oxygen-coordinated Pd species, are typically observed at higher binding energies, generally within the approximately 336–338 eV range depending on the local chemical environment and degree of metal–support interaction [41,42]. The present Pd 3d position therefore indicates that palladium was incorporated predominantly in an oxidized, electron-deficient state rather than as metallic Pd nanoparticles.

The relatively high binding energy of the Pd 3d_5_/_2_ component is also consistent with strong interaction of Pd with the oxygen-rich chemical environment provided by phosphate groups and GO surface functionalities. Both HA and GO contain potential oxygen coordination sites, and Pd introduced during hydrothermal synthesis may therefore become associated with phosphate oxygen, hydroxylated surface groups, or oxygenated carbon functionalities. Such interactions can shift Pd toward a more electron-deficient chemical state. However, the XPS data alone do not establish whether Pd^2+^ directly substitutes for Ca^2+^ within the HA lattice. Although Pd-for-Ca substitution has been demonstrated in Pd-substituted hydroxyapatite systems [36], confirmation of an equivalent substitution mechanism in the present coating would require additional crystallographic or local-structure evidence. The present data are therefore more conservatively interpreted as demonstrating predominantly oxidized and oxygen-coordinated Pd species.

This distinction between oxidized Pd and metallic Pd is particularly important for interpreting the subsequent electrochemical behavior. Metallic Pd particles incorporated into an electrically heterogeneous coating could potentially act as highly noble cathodic sites and promote localized galvanic interactions, whereas oxygen-coordinated Pd^2+^/PdO_x_-like species would influence charge transfer, surface polarity, and interactions with the surrounding HA/GO matrix differently. Furthermore, GO itself provides an electronically conductive or semiconductive carbon framework whose interfacial behavior can be modified by interaction with Pd-containing species. Consequently, the electrochemical response of the GO/Pd-containing coatings cannot be predicted simply from the individual corrosion behavior of HA, GO, or Pd.

The XPS results therefore provide an important mechanistic link between composition and electrochemical performance. The HA framework remains chemically identifiable, GO is retained with both sp^2^ carbon and oxygen-containing functionalities, and Pd exists predominantly in an oxidized chemical environment. These observations indicate that the composition-dependent corrosion behavior described later is more likely governed by local interfacial chemistry, electronic heterogeneity, coating morphology, and electrolyte-transport pathways than by formation of a completely new bulk crystalline phase.

### 3.5. Surface Morphology, Elemental Distribution, and Morphological Origin of the Corrosion Response

The surface morphologies of the as-deposited coatings are shown in Figure 5. Considerable composition-dependent differences were observed despite the preservation of the HA phase identified by XRD. These differences are particularly important for understanding the subsequent electrochemical response, because the protective effect of an HA-based coating depends not only on its phase constitution but also on its continuity, compactness, and the presence of interconnected pathways through which the electrolyte can reach the underlying metallic substrate.

The pristine HA coating exhibited a relatively coarse and heterogeneous hydrothermally grown morphology composed of agglomerated plate-like and granular HA crystallites. Local intergranular spaces were visible, although the surface remained extensively covered by calcium-phosphate deposits. Addition of 1 wt% Pd produced a comparatively smoother and more continuous coating morphology, although fine surface microcracks and isolated defects remained detectable. Increasing the Pd content to 2 wt% resulted in a finer and more uniformly distributed surface architecture, with a dense population of small HA-derived features extending across the observed area. The comparatively continuous morphology of HA-2Pd is consistent with its subsequently observed electrochemical performance and suggests that the beneficial effect of 2 wt% Pd in the GO-free HA matrix is associated, at least partly, with modification of HA nucleation and growth rather than formation of a new crystalline phase.

A markedly different morphology was obtained after GO incorporation. The HA/GO coating exhibited pronounced wrinkled and folded sheet-like features embedded within and extending across the HA deposits. These features are consistent with a GO-derived phase, as independently demonstrated by the D and G Raman bands and the oxygen-containing C 1s components detected by XPS. More importantly, the HA/GO surface was substantially more heterogeneous than the Pd-free HA coating. Large folded regions, irregular cavities, and locally open areas were observed between the GO-associated sheets and HA deposits. Thus, although GO is often reported to improve the corrosion resistance of HA-based coatings by increasing tortuosity and filling defects, the morphology obtained in the present hydrothermal system did not correspond to an ideal, uniformly dispersed two-dimensional barrier architecture.

This distinction is essential when interpreting the apparently contradictory role of GO in the present coating series. The corrosion-protective effect commonly attributed to GO requires effective dispersion and intimate integration of the nanosheets within a compact coating. Under these conditions, well-oriented and well-distributed sheets increase the diffusion path for aggressive electrolyte species and can reduce direct transport toward the metallic substrate [20,21,22,23,24,25,26,27]. In contrast, agglomerated, folded, or partially restacked GO sheets may create inter-sheet gaps, poorly mineralized regions, and interconnected interfacial channels. In such a morphology, the nominal presence of GO does not necessarily produce an effective barrier and may instead increase structural heterogeneity.

The hydrothermal precursor chemistry provides a plausible explanation for the heterogeneous GO morphology. GO carries oxygen-containing functionalities, including carboxyl and hydroxyl groups, that strongly influence its colloidal behavior in the presence of multivalent ions. Wu et al. [38] experimentally demonstrated that Ca^2+^ is substantially more effective than monovalent Na^+^ in inducing GO aggregation and attributed this behavior to divalent-cation bridging between functional groups on adjacent GO sheets. Suter and Coveney [39] further showed that under alkaline conditions, Ca^2+^ can bridge deprotonated carboxylate groups and promote both face-to-face and edge-to-edge association, producing irregular three-dimensional GO architectures. Considering that the present hydrothermal precursor contained 0.20 M Ca^2+^ and was maintained at pH ≈ 10–11, Ca^2+^-mediated partial restacking and aggregation of GO provides a plausible explanation for the folded and spatially heterogeneous sheet-like morphology observed by SEM. This mechanism is proposed on the basis of the observed morphology and established GO solution chemistry rather than as direct evidence of aggregation during the present synthesis.

The addition of Pd may further modify this interfacial environment. Strong interactions between Pd and oxygen-containing functionalities of GO have been demonstrated in Pd/GO systems [40], including modification of the local Pd–O–C environment. Although the synthesis conditions and Pd chemical state in that system differ from those of the present hydrothermal coating, the reported interaction supports the possibility that oxidized Pd species identified by XPS interact preferentially with oxygen-rich GO regions. Such interactions could locally modify HA nucleation and the organization of GO/HA interfaces.

Pd introduction further modified the GO-containing coating morphology. HA/GO-1Pd retained pronounced folded and lamellar features, with HA deposits distributed around and between the GO-associated sheets. Relatively large local depressions and interfacial voids remained evident, indicating that addition of 1 wt% Pd did not completely eliminate the microstructural heterogeneity introduced by GO. HA/GO-2Pd exhibited a more granular and apparently more filled surface at the micrometer scale, but the coating remained structurally non-uniform, with residual cavities and locally discontinuous regions. Thus, Pd addition partially modified HA deposition around the GO phase but did not convert the GO-containing coatings into morphologies equivalent to the comparatively compact Pd-modified HA surfaces.

The interaction between Pd species and GO may also contribute to this morphological evolution. Transition-metal cations are known to interact with oxygen-containing functionalities on GO, and strong Pd–GO interactions have been demonstrated in Pd/GO systems [40]. In the present hydrothermal precursor, Pd species may therefore compete with or complement Ca^2+^ interactions at oxygenated GO sites, modifying the nucleation environment for HA around the sheets. The coexistence of folded GO-derived structures and distributed Pd, together with the oxidized Pd environment identified specifically for HA/GO-2Pd by XPS, is consistent with the possibility of Pd-associated interactions within oxygen-rich GO/HA interfacial regions. However, the present data do not directly establish the molecular nature of these interactions.

EDS elemental maps of the as-deposited coatings are shown in Figure 6. Ca, P, and O were distributed over the surfaces of all six coating groups, confirming widespread formation of calcium-phosphate deposits. Pd was detected throughout the analyzed regions of the Pd-containing coatings without evidence of large micron-scale Pd-rich agglomerates. This observation is important because it indicates that the inferior electrochemical behavior of the GO/Pd coatings relative to the corresponding Pd-only coatings cannot be simply attributed to macroscopic segregation of discrete Pd particles.

The most significant EDS observation was obtained for the HA/GO coating. In addition to Ca, P, O, and C, pronounced substrate-derived Ti and Mo signals were detected. Detection of these elements through the coating indicates either locally reduced coating coverage or regions sufficiently thin and/or discontinuous for the interaction volume of the electron beam to reach the Ti30Zr5Mo substrate. This provides direct compositional support for the heterogeneous morphology observed by SEM and offers a structural explanation for the comparatively poor barrier performance of HA/GO in the electrochemical measurements.

Area-averaged (map-sum) quantification of the same maps, shown in the spectra of Figure 6, confirms that palladium is incorporated in every nominally palladium-containing coating at a level of the same order as the nominal precursor loading, and that it is distributed without segregation resolvable at the magnification used. These values are semi-quantitative: energy-dispersive X-ray spectroscopy is not a reliable quantitative method for a heavy element present at a few weight per cent in a light matrix, the analyzed volume of a porous coating is not well defined, and low-count peaks in this energy range are subject to identification ambiguity. The map-sum compositions are therefore used here only to confirm incorporation and qualitative homogeneity, and not as a determination of the retained palladium or carbon content. Interestingly, the substrate-related contribution became markedly less evident in the GO/Pd coatings, indicating that Pd addition promoted more extensive calcium-phosphate coverage relative to HA/GO alone. This observation is consistent with the improvement in electrochemical resistance obtained when 1 wt% Pd was added to HA/GO. However, the GO/Pd coatings still did not reproduce the compact interfacial architecture of the best GO-free Pd-containing coating. The remaining folded GO boundaries, cavities, and local coating heterogeneity therefore provide plausible pathways for electrolyte penetration and explain why addition of Pd only partially recovered the corrosion protection lost after GO incorporation.

This morphology–corrosion relationship also helps explain an important feature of the composition series. Pd addition alone did not adversely affect HA in a monotonic manner; indeed, HA-2Pd ultimately exhibited the lowest corrosion rate. Therefore, the inferior performance of HA/GO-Pd relative to HA-Pd cannot reasonably be attributed to Pd content alone. Instead, the results indicate that the electrochemical consequence of Pd depends strongly on the microstructural environment into which it is incorporated. Within a comparatively continuous HA matrix, 2 wt% Pd is compatible with high corrosion resistance. Within the structurally heterogeneous GO-containing matrix, the same Pd level is associated with a substantially different electrochemical response.

At the same time, SEM morphology alone does not fully explain the difference between HA/GO-1Pd and HA/GO-2Pd. Although HA/GO-2Pd appears relatively filled at the micrometer scale, its electrochemical performance is inferior to that of HA/GO-1Pd. This discrepancy indicates that surface compactness visible by SEM is only one component of the corrosion mechanism. The oxidation state of Pd, local GO–Pd interactions, nanoscale defects, electrolyte transport along buried sheet/coating interfaces, and the resulting charge-transfer heterogeneity must also be considered. These effects are examined quantitatively using the EIS and potentiodynamic polarization results in Section 3.6.

### 3.6. Electrochemical Corrosion Behavior

#### 3.6.1. Electrochemical Impedance Spectroscopy

The electrochemical impedance behavior of the HA, HA-1Pd, HA-2Pd, HA/GO, HA/GO-1Pd, and HA/GO-2Pd coatings in Hank’s solution at 37 °C is presented in Figure 7. The impedance spectra were interpreted using the two-time-constant equivalent electrical circuit Rs(Qc(Rcoat(QdlRct))), in which Rs represents the solution resistance, and Qc and Rcoat describe the non-ideal capacitive response and resistance of the outer-coating layer, respectively, while Qdl and Rct represent the interfacial double-layer response and charge-transfer resistance at electrochemically accessible regions of the coating/substrate interface.

The Bode modulus plots (Figure 7A) revealed clear composition-dependent differences in the low-frequency impedance response. The HA coating exhibited the highest low-frequency impedance modulus, approaching approximately 1.5 × 10^4^ Ω·cm^2^ at 0.01 Hz, indicating an effective barrier against electrolyte penetration. HA-2Pd exhibited a similarly high overall impedance response, whereas HA-1Pd and HA/GO-1Pd showed intermediate behavior. In contrast, HA/GO produced the lowest low-frequency impedance among the coating series, demonstrating that incorporation of GO alone did not provide the barrier enhancement often associated with well-dispersed graphene-derived phases.

The phase-angle plots (Figure 7C) provide additional evidence for differences in coating architecture. HA exhibited a relatively broad and pronounced capacitive response, consistent with a comparatively homogeneous barrier layer. Following incorporation of Pd and/or GO, two partially overlapping relaxation processes became more evident, indicating increased separation between the response of the outer coating and that of electrochemically accessible regions near the underlying interface. The emergence of these distinguishable time constants is consistent with the increased structural and chemical complexity of the modified coatings.

The Nyquist plots (Figure 7B) further illustrate this behavior. HA showed the largest and most complete capacitive arc, whereas HA-2Pd maintained a comparatively large impedance response. The Pd-containing and GO/Pd-containing coatings exhibited intermediate semicircular responses, while HA/GO produced the smallest arc and a more open low-frequency trajectory. Notably, the HA/GO curve also exhibited a distinctly inclined low-frequency tail, approaching a diffusion-like response. The latter behavior is consistent with enhanced electrolyte transport through a structurally heterogeneous coating and agrees with the folded GO morphology and increased substrate-associated Ti/Mo signal observed by SEM–EDS. Unlike the other coatings, the inclined low-frequency response of HA/GO may indicate an additional mass-transport contribution within the heterogeneous coating rather than a predominantly capacitive interfacial response.

The equivalent circuit Rs(Qc(Rcoat(Qdl·Rct))) was retained for all six coatings, including HA/GO, and this choice requires justification. A single common circuit is a prerequisite for comparing fitted parameters across a composition series; introducing a diffusion element for one composition alone would render its resistive parameters incommensurable with those of the remaining five. More importantly, the two constant-phase elements already describe a distributed, non-ideal response of precisely the kind produced by restricted electrolyte transport through a heterogeneous layer. The low inner-interface exponent obtained for HA/GO, ndl = 0.417, is the quantitative expression of that distribution rather than an artifact of an incomplete circuit, and it is itself one of the principal results of this section. The goodness of fit supports the choice: the chi-squared value obtained for HA/GO, 5.09 × 10^−5^, is of the same order as, and in fact lower than, those obtained for several of the remaining coatings, which indicates that the retained circuit reproduces the measured spectrum without systematic deviation. Accordingly, the inclined low-frequency tail of HA/GO is treated in this work strictly as qualitative evidence of enhanced electrolyte transport through a structurally heterogeneous coating. No Warburg coefficient, diffusion coefficient, or diffusion-layer thickness is extracted from it, and no quantitative mass-transport claim is made. Measurements extending to lower frequencies, together with a formal comparison of alternative circuits, would be required before a diffusion element could be justified for this composition, and this is identified as further work in Section 3.10.

Equivalent-circuit fitting parameters are summarized in Table 4. For clarity, the resistance terms are discussed here as Rcoat, Rct, and the total resistance (Rtot = Rcoat + Rct), thereby avoiding confusion with the Stern–Geary polarization resistance obtained independently from the potentiodynamic polarization measurements.

Based on the mean Rtot values, the descriptive order was HA (16.52 ± 0.40 kΩ·cm^2^) > HA-2Pd (15.73 ± 0.13 kΩ·cm^2^) > HA/GO-1Pd (14.97 ± 0.20 kΩ·cm^2^) > HA-1Pd (14.79 ± 0.37 kΩ·cm^2^) > HA/GO-2Pd (11.73 ± 0.30 kΩ·cm^2^) > HA/GO (8.50 ± 0.15 kΩ·cm^2^).

This ranking demonstrates that the influence of GO and Pd was not additive in a simple monotonic manner. Pristine HA already provided an effective electrochemical barrier. Incorporation of 1 wt% Pd produced a modest reduction in the mean total EIS resistance, whereas increasing Pd to 2 wt% restored the response to a value close to that of pristine HA. In contrast, GO incorporation alone reduced the mean total resistance by approximately one-half relative to HA, indicating that the GO-containing morphology generated under the present hydrothermal conditions was substantially less effective as a corrosion barrier.

The fitted constant-phase element (CPE) exponents provide further insight into this behavior. The inner-interface exponent was highest for HA (n = 0.915), indicating a response relatively close to ideal capacitive behavior. HA/GO exhibited by far the lowest value (n = 0.417), demonstrating a much broader distribution of interfacial relaxation times and therefore a considerably more heterogeneous electrochemical interface. Addition of Pd increased the exponent in the GO-containing series to 0.612 and 0.626 for HA/GO-1Pd and HA/GO-2Pd, respectively. Thus, Pd partially reduced the extreme interfacial heterogeneity generated by GO alone, consistent with the improved surface coverage observed by SEM–EDS.

By contrast, the outer-layer CPE exponents (nc) varied only within a relatively narrow range of approximately 0.790–0.833 (Table 5). This comparatively small variation indicates that the major electrochemical differences among the coatings were not controlled solely by the non-ideality of the outermost coating surface. Rather, the larger changes in Rct and in the inner-interface CPE exponent indicate that electrolyte access to buried coating/substrate regions and the electrochemical nature of those regions played the dominant role.

This distinction becomes particularly important when comparing the two GO/Pd coatings. Increasing the Pd content from 1 to 2 wt% increased the representative Rcoat from 638.4 to 686.2 Ω·cm^2^, suggesting that the apparent resistance of the outer-coating layer was not degraded at the higher Pd content. Nevertheless, the representative Rct decreased substantially from 14.27 to 11.12 kΩ·cm^2^. Consistent with this change, the mean total resistance decreased from 14.97 ± 0.20 kΩ·cm^2^ for HA/GO-1Pd to 11.73 ± 0.30 kΩ·cm^2^ for HA/GO-2Pd.

This result indicates that the deterioration observed for HA/GO-2Pd cannot be explained simply by poorer macroscopic coating coverage. Instead, once electrolyte penetrated through accessible pathways in the GO-containing architecture, the electrochemical processes occurring at the inner interface became more favorable at the higher Pd loading. This represents one of the most important findings of the present study.

GO is frequently reported to improve corrosion protection when its nanosheets are uniformly dispersed within a compact coating because the two-dimensional sheets increase the tortuosity of ionic transport pathways [20,21,22,23,24,25,26,27]. The present results demonstrate that this mechanism is highly dependent on the manner in which GO is incorporated. As discussed in Section 3.5, the Ca^2+^-rich alkaline hydrothermal environment can promote partial GO restacking and aggregation through divalent-ion bridging [38,39]. The resulting folded GO domains and HA/GO interfacial boundaries provide pathways through which electrolyte can penetrate more readily than through a uniformly compact HA matrix. This accounts for the exceptionally low Rct, low CPE exponent, and poor overall impedance response of HA/GO.

Addition of 1 wt% Pd partially compensated for this disadvantage. HA/GO-1Pd exhibited markedly higher representative Rcoat and Rct values than HA/GO, consistent with the more extensive calcium-phosphate coverage observed by SEM–EDS. One plausible interpretation is that Pd-associated interactions at oxygen-containing GO regions modified the local HA nucleation environment and reduced some of the large-scale discontinuities present in HA/GO. Strong Pd–GO interactions have previously been demonstrated in oxygen-functionalized graphene systems [40], although the chemical state and synthesis route used in those studies differ from the present hydrothermal system.

The behavior at 2 wt% Pd is more complex. Because HA/GO-2Pd exhibited a higher Rcoat than HA/GO-1Pd but a lower Rct, the deterioration at the higher Pd level is more consistent with an interfacial electrochemical effect than with a simple loss of surface compactness. XPS analysis of HA/GO-2Pd indicated that Pd was predominantly present in an oxidized and oxygen-coordinated chemical environment rather than as metallic Pd^0^. The higher nominal Pd loading may therefore alter the density and distribution of Pd-associated sites within oxygen-rich GO/HA interfacial regions, potentially affecting local electronic structure, surface polarity, and charge-transfer kinetics. At the same time, the GO-derived carbon framework provides electronically distinct regions embedded within an ionic calcium-phosphate matrix. Increasing the density of Pd-containing sites within this heterogeneous network may therefore generate a larger number of electrochemically active interfacial regions once electrolyte reaches the buried coating structure.

This interpretation does not require the presence of large metallic Pd particles or classical macroscopic galvanic coupling, neither of which was demonstrated by the present characterization. Rather, the data support a more localized mechanism in which the combination of GO-associated transport pathways and Pd-modified interfacial chemistry controls charge transfer. The decrease in Rct from HA/GO-1Pd to HA/GO-2Pd is consistent with a composition-dependent change in buried interfacial electrochemistry.

Interestingly, the same increase from 1 to 2 wt% Pd produced the opposite trend in the GO-free HA matrix. Although Rcoat changed only moderately, Rct increased from 14.30 kΩ·cm^2^ for HA-1Pd to 15.39 kΩ·cm^2^ for HA-2Pd. Thus, the electrochemical effect of Pd depends strongly on whether GO is present. Within the HA matrix alone, the higher Pd level is compatible with improved interfacial resistance, whereas within the GO-containing architecture the same increase in Pd decreases Rct. This contrast provides strong evidence that the corrosion response is governed by GO–Pd–HA interfacial interactions rather than by Pd concentration alone.

#### 3.6.2. Potentiodynamic Polarization Behavior

The potentiodynamic polarization curves are presented in Figure 8, and the corresponding electrochemical parameters are summarized in Table 6. The polarization measurements generally support the composition-dependent trends obtained from EIS, although the exact ranking differs because EIS probes small-amplitude near-equilibrium behavior, whereas potentiodynamic polarization drives the coating/substrate system through a substantially wider potential range. The quantitative PDP parameters are reported as mean ± standard deviation for three independently prepared specimens (n = 3).

HA-2Pd exhibited the lowest corrosion current density of the coating series, with an icorr of 1.46 ± 0.02 µA·cm^−2^, and consequently the lowest calculated corrosion rate of 0.0127 ± 0.0002 mm·year^−1^. It also exhibited the most positive corrosion potential, Ecorr = +0.0438 ± 0.0015 V. These results identify 2 wt% Pd as the most favorable Pd content within the GO-free HA series in terms of potentiodynamic corrosion behavior.

Pristine HA exhibited an icorr of 1.95 ± 0.04 µA·cm^−2^ and a corrosion rate of 0.0169 ± 0.0003 mm·year^−1^. Incorporation of 1 wt% Pd increased icorr to 3.14 ± 0.07 µA·cm^−2^ and the corrosion rate to 0.0273 ± 0.0006 mm·year^−1^, whereas increasing the Pd level to 2 wt% markedly improved the corrosion response. The non-monotonic behavior therefore indicates that the effect of Pd cannot be explained solely by its nominal concentration or the noble character of elemental Pd but is instead associated with composition-dependent modification of coating nucleation and interfacial electrochemistry.

Among the GO-containing coatings, HA/GO exhibited the highest corrosion current density, 4.21 ± 0.07 µA·cm^−2^, together with the highest corrosion rate, 0.0366 ± 0.0006 mm·year^−1^, confirming that GO alone provided the poorest corrosion protection within the investigated coating series. This result agrees with its comparatively low EIS resistance, low interfacial CPE exponent, heterogeneous SEM morphology, and pronounced Ti/Mo substrate contribution detected by EDS.

Addition of 1 wt% Pd to HA/GO substantially improved the polarization response. The corrosion current density decreased from 4.21 ± 0.07 to 2.15 ± 0.04 µA·cm^−2^, while the corrosion rate decreased from 0.0366 ± 0.0006 to 0.0187 ± 0.0002 mm·year^−1^. Thus, incorporation of 1 wt% Pd recovered a substantial fraction of the corrosion resistance lost following GO addition.

However, increasing Pd from 1 to 2 wt% within the GO-containing system again deteriorated the electrochemical response. HA/GO-2Pd exhibited an icorr of 2.91 ± 0.06 µA·cm^−2^ and a corrosion rate of 0.0253 ± 0.0005 mm·year^−1^. This deterioration relative to HA/GO-1Pd mirrors the decrease in the representative EIS-derived Rct from approximately 14.27 to 11.12 kΩ·cm^2^, reinforcing the interpretation that higher Pd incorporation within the GO-containing architecture promotes greater electrochemical activity at buried interfaces despite maintaining or slightly increasing the resistance of the outer-coating layer.

The corrosion-potential response further demonstrates that Pd does not act identically in the two coating matrices. In the GO-free series, Ecorr shifted progressively in the positive direction from −0.0856 ± 0.0032 V for HA to −0.0583 ± 0.0021 V for HA-1Pd and +0.0438 ± 0.0015 V for HA-2Pd. In contrast, the GO-containing series did not exhibit a monotonic potential shift: Ecorr changed from +0.0321 ± 0.0022 V for HA/GO to −0.0373 ± 0.0018 V for HA/GO-1Pd and subsequently to +0.0124 ± 0.0020 V for HA/GO-2Pd. The absence of a simple monotonic relationship in the GO-containing coatings further supports a composition-dependent GO–Pd–HA interfacial effect.

It should also be noted that the Stern–Geary polarization resistance obtained from the potentiodynamic curves does not rank the samples identically to the EIS-derived resistance values. This difference is expected because the two techniques probe fundamentally different electrochemical regimes and because the Stern–Geary resistance is influenced by the measured anodic and cathodic Tafel slopes. Consequently, the EIS and polarization results should be regarded as complementary rather than interchangeable. Therefore, the identification of an optimum composition should not rely on a single electrochemical parameter. In the present system, HA exhibited the highest mean EIS resistance, whereas HA-2Pd showed the lowest icorr and corrosion rate, indicating that HA-2Pd improved the polarization response without exceeding pristine HA in terms of total impedance resistance.

Taken together, the EIS and potentiodynamic polarization results demonstrate that GO and Pd do not exert independent or simply additive effects on the corrosion behavior of the hydrothermally deposited HA coatings. GO alone produced the least protective coating because its incorporation generated a heterogeneous architecture containing more accessible electrolyte-transport pathways. Addition of 1 wt% Pd partially restored coating continuity and interfacial resistance. Increasing Pd to 2 wt% further improved corrosion resistance in the GO-free HA matrix but decreased Rct and increased the corrosion rate when GO was present. This contrasting behavior demonstrates that the optimum Pd concentration is matrix dependent and that the electrochemical performance of HA/GO/Pd coatings is governed by the coupled effects of GO organization, Pd-associated interfacial chemistry, HA nucleation, and buried interfacial charge transfer.

### 3.7. Post-Corrosion Surface Morphology and Compositional Evolution

Figure 9 shows the surface morphologies of the coatings after potentiodynamic polarization. Despite the relatively severe potential excursion imposed during polarization, the characteristic hydrothermally developed coating architecture remained recognizable in all six compositions. No evidence of widespread coating spallation or complete exposure of large areas of the Ti30Zr5Mo substrate was observed at the investigated magnification. This indicates that the differences in electrochemical performance discussed in Section 3.6 were associated predominantly with localized electrolyte penetration, interfacial charge transfer, and composition-dependent dissolution processes rather than catastrophic detachment of the entire coating layer.

This observation is particularly relevant to the poor electrochemical performance of HA/GO. Both EIS and PDP identified HA/GO as the least protective composition, yet the post-polarization surface still retained substantial calcium-phosphate coverage. Therefore, its high corrosion current density and low impedance cannot be attributed simply to complete removal of the coating. Instead, the results support the mechanism proposed from the as-deposited SEM–EDS observations: folded or heterogeneously incorporated GO domains created locally accessible transport pathways that allowed the electrolyte to reach electrochemically active regions beneath an otherwise macroscopically retained coating.

The Pd-containing coatings similarly retained their overall coating morphology after polarization. The continued presence of coating material on HA-1Pd and HA-2Pd is consistent with the comparatively high EIS resistances obtained for these compositions, particularly HA-2Pd. Importantly, the superior corrosion performance of HA-2Pd was therefore achieved without evidence of extensive coating loss during polarization, supporting the interpretation that the 2 wt% Pd modification produced a comparatively favorable combination of coating continuity and interfacial electrochemical resistance in the GO-free HA matrix.

The GO/Pd coatings also remained visibly covered after polarization, despite the composition-dependent difference between HA/GO-1Pd and HA/GO-2Pd observed electrochemically. This finding reinforces an important conclusion from the EIS analysis. The deterioration from 1 to 2 wt% Pd in the GO-containing series cannot be explained solely by macroscopic coating removal or gross structural failure. Instead, the lower Rct and higher icorr of HA/GO-2Pd point toward changes occurring within electrochemically accessible GO–Pd–HA interfaces beneath or within the retained coating architecture.

Post-polarization point-EDS spectra are presented in Figure 10, and the corresponding Ca, P, and Pd contents are summarized in Table 7. Ca and P remained detectable as the dominant coating-related elements in every composition, confirming retention of the calcium-phosphate phase after electrochemical exposure. Pd was also detected in each Pd-containing specimen after polarization. Thus, the electrochemical test did not result in complete removal of the Pd-containing coating phase or obvious selective elimination of Pd from the analyzed surface regions.

The calculated local Ca/P atomic ratios nevertheless revealed substantial composition-dependent differences after polarization. The measured ratios were 1.97 for HA, 2.04 for HA-1Pd, and 2.21 for HA-2Pd in the GO-free series. In comparison, the GO-containing coatings exhibited values of 1.77 for HA/GO, 1.72 for HA/GO-1Pd, and 1.44 for HA/GO-2Pd.

These values should be interpreted cautiously because EDS provides a local and semi-quantitative analysis of the post-corrosion surface and does not represent the bulk stoichiometry of the coating. Furthermore, the measurements were obtained after potentiodynamic polarization, when dissolution, ion exchange, and possible surface reprecipitation may already have modified the local composition. Consequently, deviation from the theoretical Ca/P atomic ratio of stoichiometric HA (1.67) should not be used alone to infer the initial phase composition of the as-deposited coatings.

Nevertheless, the contrasting evolution of the two coating families is noteworthy. In the GO-free series, increasing Pd content was accompanied by progressively Ca-rich local post-polarization compositions, with HA-2Pd exhibiting the highest measured Ca/P ratio. This same composition displayed the lowest icorr and corrosion rate and one of the highest EIS-derived total resistances. Although the EDS data cannot establish the mechanism responsible for this association, they indicate that the electrochemically exposed surface of HA-2Pd evolved differently from those of the GO-containing coatings.

The opposite trend occurred within the GO-containing series. The Ca/P ratio decreased from 1.77 for HA/GO to 1.72 for HA/GO-1Pd and further to 1.44 for HA/GO-2Pd. The pronounced reduction for HA/GO-2Pd is particularly noteworthy because this coating also exhibited a lower Rct and a higher corrosion rate than HA/GO-1Pd. Taken together, these observations suggest that increasing Pd content within the GO-containing matrix altered the local dissolution/reprecipitation behavior of the calcium-phosphate surface.

A Ca/P ratio below the nominal HA value after polarization may arise from preferential removal of Ca-containing species and/or development of a relatively P-rich residual surface. The present point-EDS measurements cannot distinguish unequivocally between these processes, and therefore preferential Ca dissolution should be regarded as a plausible interpretation rather than a directly demonstrated mechanism. However, the particularly low value obtained for HA/GO-2Pd is consistent with the electrochemical evidence that the higher Pd loading generated a more reactive buried interfacial environment when GO was present.

This provides an additional connection between the morphological, chemical, and electrochemical results. GO alone generated a heterogeneous coating architecture that facilitated electrolyte access. Addition of 1 wt% Pd improved coating coverage and substantially increased both Rcoat and Rct, thereby reducing the corrosion rate. Increasing Pd to 2 wt% further increased the outer-layer Rcoat, demonstrating that the external coating barrier itself was not necessarily poorer. However, the simultaneous decrease in Rct, increase in icorr, and reduction of the post-polarization Ca/P ratio to 1.44 indicate that the internal electrochemical environment became less stable.

The combined results therefore support a two-level corrosion mechanism for the GO/Pd coatings. At the first level, the organization of GO within the hydrothermal HA matrix determines electrolyte-access pathways. Ca^2+^-mediated restacking and heterogeneous HA nucleation can generate buried interfaces through which physiological electrolyte progressively penetrates. At the second level, once these pathways become electrochemically accessible, the local chemistry of the GO–Pd–HA interface controls charge-transfer and dissolution kinetics. At 1 wt% Pd, improved HA coverage appears to compensate substantially for the structural disadvantage introduced by GO. At 2 wt% Pd, however, the higher nominal Pd loading may alter the density and distribution of Pd-associated sites within the oxygen-rich GO/HA interfacial network, thereby favoring increased interfacial electrochemical activity despite the persistence of a macroscopically intact outer coating.

This mechanism also explains why GO should not be considered intrinsically detrimental on the basis of the present results. The conventional corrosion-barrier effect of GO depends on obtaining sufficiently dispersed and well-integrated nanosheets. The present hydrothermal Ca-rich environment produced a different microstructural condition, in which GO-associated interfacial heterogeneity outweighed the expected tortuous-path benefit. Pd subsequently modified this balance in a concentration-dependent manner. Thus, the decisive variable is not simply the presence or absence of GO, but the organization and chemistry of the GO–Pd–HA interface.

Based on the combined SEM–EDS, XPS, EIS, PDP, and post-polarization compositional results, the proposed composition-dependent corrosion mechanism is summarized in Scheme 1. The model highlights that the corrosion response is governed not simply by the nominal GO or Pd content, but by GO organization, Pd-associated interfacial chemistry, electrolyte-access pathways, and charge-transfer processes at buried GO–Pd–HA interfaces.

Overall, the post-polarization SEM–EDS analysis confirms that the composition-dependent corrosion response occurred while the HA-based coatings remained substantially retained on the substrates. The results therefore reinforce the conclusion that localized electrolyte transport and buried interfacial electrochemistry, rather than catastrophic coating failure, govern the differences among the six compositions. In particular, the combination of reduced Rct, increased corrosion rate, and the markedly lower post-polarization Ca/P ratio of HA/GO-2Pd provides complementary evidence that increasing Pd from 1 to 2 wt% changes the electrochemical stability of the GO-containing HA interface.

### 3.8. In Vitro Cellular Response

The cellular response of A7R5 cells to the uncoated β-Ti30Zr5Mo substrate and the six HA-based coating compositions was evaluated by phase-contrast microscopy after 24 h of culture. Representative phase-contrast micrographs are presented in Figure 11, while the corresponding cell-density values are summarized in Table 8. Quantitative cell-density results are reported as mean ± standard deviation from three independently prepared specimens (n = 3).

A pronounced increase in surface-associated cell density was observed for all HA-based coatings compared with the uncoated substrate. The uncoated β-Ti30Zr5Mo control exhibited a cell density of 6502 ± 175 cells/cm^2^, whereas the HA coating supported 16,058 ± 420 cells/cm^2^, corresponding to 247.0% of the control value. This increase indicates that hydrothermal HA deposition generated a surface favorable for early A7R5 cell attachment and/or retention under the investigated 24 h culture conditions.

Pd incorporation was accompanied by a progressive increase in cell density within the GO-free coating series. The measured values increased from 16,058 ± 420 cells/cm^2^ for HA to 19,107 ± 503 cells/cm^2^ for HA-1Pd and 20,513 ± 501 cells/cm^2^ for HA-2Pd. These values correspond to 247.0, 293.9, and 315.5% of the uncoated control, respectively. Thus, incorporation of Pd did not produce an evident adverse effect on the short-term A7R5 cellular response, and the 2 wt% Pd composition exhibited the highest descriptive cell-density value within the GO-free series.

GO incorporation also resulted in a comparatively high cellular response. HA/GO exhibited a cell density of 18,986 ± 390 cells/cm^2^**,** corresponding to 292.0% of the uncoated control and exceeding the mean value obtained for pristine HA. Notably, HA/GO supported a high number of surface-associated cells despite exhibiting the poorest electrochemical corrosion resistance among the six coating compositions. This contrast demonstrates that the factors governing electrochemical barrier performance and short-term cellular response were not identical. The scientific content, structure, panel organization, and all labels and values of this scheme were conceived and specified by the authors. The final artwork was rendered with the assistance of a generative artificial intelligence image-generation tool, GPT 5.6, and the rendered scheme was subsequently checked against the experimental data and corrected by the authors, who take full responsibility for its scientific content and accuracy. The scheme presents no experimental data that are not reported and supported elsewhere in this manuscript.

A similar progressive increase with Pd content was observed in the GO-containing series. Cell density increased from 18,986 ± 390 cells/cm^2^ for HA/GO to 20,025 ± 458 cells/cm^2^ for HA/GO-1Pd and reached 21,471 ± 516 cells/cm^2^ for HA/GO-2Pd. These values correspond to 292.0, 308.0, and 330.2% of the uncoated control, respectively. HA/GO-2Pd therefore exhibited the highest mean cell density of the entire coating series.

At each corresponding Pd level, the GO-containing coating exhibited a higher mean cell-density value than its GO-free counterpart. HA/GO exceeded HA, HA/GO-1Pd exceeded HA-1Pd, and HA/GO-2Pd exceeded HA-2Pd. These observations indicate a consistent descriptive tendency toward greater surface-associated cell density in the GO-containing groups. One-way ANOVA indicated a significant overall effect of coating composition on A7R5 cell density (*p* < 0.05).

The cellular and electrochemical results therefore reveal a clear functional divergence. HA-2Pd provided the most favorable corrosion response, whereas HA/GO-2Pd produced the highest A7R5 cell density. Conversely, HA/GO exhibited the lowest EIS resistance and the highest corrosion rate but still supported substantially greater cell density than the uncoated substrate and a higher mean value than pristine HA. Thus, the structural features that impaired the electrochemical barrier effectiveness of the GO-containing coating did not translate into an unfavorable short-term A7R5 response.

This behavior is consistent with the different surface requirements governing corrosion protection and cell–material interactions. Corrosion resistance is strongly dependent on coating continuity, electrolyte-transport pathways, and buried interfacial charge transfer, whereas early cell attachment is governed primarily by the outermost surface chemistry, topography, and micro/nanostructure encountered by the cells. Consequently, a heterogeneous GO-containing coating may contain buried pathways that facilitate electrolyte penetration while simultaneously presenting an outer surface compatible with cell attachment.

XPS analysis of the HA/GO-2Pd coating indicated that Pd was present predominantly in an oxidized and oxygen-coordinated chemical environment rather than as metallic Pd^0^. The high cell density observed for HA/GO-2Pd therefore indicates that this Pd-containing surface chemistry did not produce an evident adverse effect on A7R5 attachment during the 24 h exposure period. Nevertheless, because the present assay quantified surface-associated cell density rather than metabolic activity, the results should not be interpreted as a direct measurement of cell viability or as evidence of long-term cytocompatibility.

Accordingly, the values reported in Table 8 are expressed as relative cell density rather than “cell viability.” Phase-contrast cell counting provides quantitative information on the number of cells associated with the specimen surface but does not directly assess mitochondrial activity, membrane integrity, apoptosis, or longer-term proliferative behavior. The present experiment therefore demonstrates a favorable short-term cellular response to all six HA-based coatings, with HA/GO-2Pd exhibiting the highest surface-associated cell density under the investigated conditions.

Overall, the cellular results reinforce the multifunctional nature of the coating system. HA-2Pd provided the strongest electrochemical protection, whereas HA/GO-2Pd generated the greatest short-term A7R5 cell-density response. Optimization of HA/GO/Pd coatings should therefore consider electrochemical and biological performance simultaneously rather than assuming that the composition maximizing one response will necessarily maximize the other.

### 3.9. Bacterial Bioluminescence Response

The response of bioluminescent *Staphylococcus aureus* Xen36 to the six coating compositions was evaluated by IVIS imaging, as shown in Figure 12, while the corresponding total radiant flux values are presented in Figure 13. Because the images in Figure 12 are displayed using different color-scale ranges, direct visual comparison of image brightness across differently scaled panels should be made cautiously. Accordingly, the quantitative total radiant flux values shown in Figure 13 were used as the primary basis for comparison among the coating compositions.

A clear composition-dependent response was observed. HA, HA-1Pd, HA-2Pd, and HA/GO exhibited comparatively low bioluminescent signals, generally on the order of a few ×10^6^ photons/s. In contrast, the two GO/Pd-containing coatings produced substantially greater total radiant flux. HA/GO-1Pd exhibited the highest bacterial bioluminescent signal, while HA/GO-2Pd also showed a markedly elevated response relative to the four remaining coating compositions. The difference was approximately one order of magnitude in the most pronounced comparison.

The simultaneous presence of GO and Pd did not suppress the Xen36 bioluminescent response under the conditions of the present assay. Importantly, neither GO nor Pd alone produced the strong increase observed for the combined GO/Pd coatings. HA/GO remained within the comparatively low-signal group, as did both HA-1Pd and HA-2Pd. The elevated response therefore appears to be associated specifically with the chemical and/or interfacial environment generated when GO and Pd were incorporated together rather than with either component individually.

The bacterial response is particularly noteworthy when compared with the electrochemical and cellular results. Addition of 1 wt% Pd to HA/GO substantially improved the corrosion behavior relative to HA/GO alone, while increasing Pd content within both coating families produced comparatively high A7R5 cell densities. Nevertheless, the GO/Pd combinations generated the highest Xen36 bioluminescent responses. Thus, the biological functions investigated in the present study did not evolve in parallel: a coating composition that was favorable for mammalian cell attachment was not necessarily favorable for suppression of the bacterial reporter response.

The observation also cautions against assuming that the antimicrobial properties reported independently for graphene-derived materials or noble-metal-containing surfaces will necessarily be retained when the two components are incorporated within a hydrothermal HA matrix. The biological behavior of a multicomponent coating is determined by the actual chemical state, surface accessibility, spatial distribution, and interfacial organization of its constituents rather than their nominal presence alone. For HA/GO-2Pd, XPS indicated that Pd was present predominantly in an oxidized and oxygen-coordinated environment rather than as metallic Pd^0^. Together with the SEM and electrochemical observations, this finding illustrates that the surface and interfacial environment encountered by bacteria in the GO/Pd-containing coatings differs substantially from that of isolated GO sheets or surfaces bearing exposed metallic Pd nanoparticles.

The finding that HA/GO-1Pd produced a greater bioluminescent response than HA/GO-2Pd further indicates that the bacterial response was not simply proportional to nominal Pd concentration. This non-monotonic behavior resembles the broader composition dependence observed in the electrochemical measurements and suggests that the organization and chemical state of the GO–Pd–HA interface may be more important than the total amount of Pd incorporated into the precursor solution.

However, the IVIS results must be interpreted according to the specific nature of the Xen36 assay. Bioluminescence reflects the activity of the bacterial lux reporter system and therefore depends on the presence of metabolically active bacteria as well as the biochemical conditions required for light production. Total radiant flux is consequently a useful comparative indicator of bacterial activity/load under identical experimental conditions, but it is not equivalent to a direct colony-forming-unit count.

For this reason, the increased signal of the GO/Pd coatings should not be described as definitive evidence that these coatings caused a corresponding one-to-one increase in bacterial cell number. Differences in the local physicochemical environment of the coating could potentially influence bacterial metabolic state and, consequently, reporter output. In particular, the GO/Pd surfaces differ from the other compositions in carbon chemistry, Pd coordination state, surface morphology, and interfacial redox characteristics. The present dataset does not allow these effects to be separated from differences in bacterial abundance.

Accordingly, the most defensible conclusion from the present experiment is that GO/Pd co-incorporation did not suppress the Xen36 bioluminescent response and instead produced a substantially higher bacterial reporter signal than either GO or Pd modification alone. Confirmation of whether this elevated signal corresponds directly to increased viable bacterial numbers would require an orthogonal quantitative method, such as CFU enumeration or an appropriate live/dead bacterial assay.

This finding has important implications for the multifunctional optimization of the coating system. HA-2Pd provided the best overall corrosion performance while maintaining a favorable mammalian cellular response and a comparatively low Xen36 signal. In contrast, HA/GO-2Pd generated the highest A7R5 cell density but simultaneously exhibited an elevated bacterial bioluminescent response and inferior corrosion resistance compared with HA-2Pd. Therefore, maximizing one biological response cannot be assumed to optimize the overall implant-coating performance.

Taken together, the bacterial-response assessment reveals a clear functional trade-off within the six-member composition series. GO and Pd co-incorporation can modify electrochemical behavior and support high mammalian cell density, but under the present processing conditions it does not produce a corresponding reduction in the Xen36 bioluminescent response. The results therefore identify control of the GO–Pd interfacial state and bacterial response as important targets for further optimization of these multifunctional HA-based coatings.

### 3.10. Limitations of the Present Study and Directions for Further Work

Several limitations of the present work should be stated explicitly, because they define the boundaries within which the proposed mechanisms are supported by direct evidence. Coating thickness, surface roughness, porosity, and adhesion strength were not measured quantitatively in this study. Differences in coverage and continuity between the compositions were therefore inferred indirectly, from the relative intensity of the substrate reflections in the diffraction patterns, from the substrate-derived Ti and Mo signals detected by energy-dispersive X-ray spectroscopy, and from the constant-phase-element exponents obtained by impedance fitting. These indicators are mutually consistent, but they cannot separate the intrinsic chemical contribution of graphene oxide and palladium from the contribution of the different morphologies that these additives produce. Cross-sectional thickness measurement, contact or optical profilometry, and scratch or pull-off adhesion testing are required before such a separation can be made, and these measurements are necessary before such a separation can be claimed.

The palladium and graphene oxide contents quoted throughout the text are nominal precursor values. Area-averaged (map-sum) energy-dispersive X-ray analysis of the as-deposited coatings, shown in Figure 6, indicates that palladium is incorporated at a level of the same order as the nominal loading and that it is distributed without resolvable segregation at the magnification used, but energy-dispersive X-ray spectroscopy is not a reliable quantitative method for a light matrix containing a heavy element at this concentration. Inductively coupled plasma optical emission spectrometry performed on dissolved coatings would provide the actual retained palladium content, and thermogravimetric or elemental analysis would provide the retained carbon content; neither analysis was available for the present specimen set.

X-ray photoelectron spectroscopy was performed only on the HA/GO-2Pd coating, which was selected as the composition combining graphene oxide with the highest nominal palladium loading. The chemical state of palladium reported here therefore applies to that composition, and the interpretation of the difference between 1 and 2 wt% palladium and between the GO-free and GO-containing series rests on the electrochemical and morphological evidence rather than on a direct comparison of Pd 3d spectra. High-resolution Pd 3d and C 1s spectra of the complete composition series are required before the chemical state of palladium can be compared across the series, and this comparison is identified here as necessary further work.

The restacking and aggregation of graphene oxide sheets promoted by Ca^2+^ is presented in this work as a plausible and internally consistent explanation of the folded, sheet-like domains observed by scanning electron microscopy, of the low interfacial constant-phase-element exponent of HA/GO, and of the low-angle diffraction feature of the GO-containing coatings. It is not, however, demonstrated directly. Transmission electron microscopy of coating cross-sections, atomic force microscopy of the sheet topography, and Raman mapping of the spatial distribution of the D and G bands are required to establish the arrangement of the sheets within the apatite matrix, and the mechanism is described throughout the text as proposed rather than as established.

The electrochemical comparison was made within the coating series and does not include the uncoated β-Ti30Zr5Mo alloy, so the absolute protective contribution of the coatings relative to the bare substrate is not quantified here. The uncoated alloy was, however, included as the reference in the cellular experiments. Adding the bare alloy to the impedance and polarization measurements is necessary to express the protective efficiency of each coating, and this reference measurement is necessary before a protective efficiency can be reported.

Finally, the biological evaluation was deliberately restricted in scope. A7R5 rat aortic smooth-muscle cells were used as a comparative mammalian cell–surface response model for short-term attachment and surface-associated cell density, and they are not a model of osteogenic differentiation; the normalized values reported in Section 3.8 are accordingly interpreted as relative cell densities and not as measures of metabolic viability or of bone-forming capacity. An osteoblastic cell line, a live/dead assay, and a metabolic assay over longer culture periods are required before osteointegration can be addressed. Similarly, bioluminescence from *Staphylococcus aureus* Xen36 reports the metabolic activity of the luminescent population and is not a direct count of viable adherent bacteria. The bacterial results are therefore presented as relative bioluminescent responses, and colony-forming-unit counting, live/dead fluorescence imaging, and quantification of biofilm biomass are required before any claim of antibacterial efficacy can be made for these coatings. The bacterial experiment also did not include the uncoated alloy or a positive antibacterial control, so the bioluminescent responses are comparable within the coating series but cannot be referred to an absolute scale. Finally, the coatings were not re-characterized after sterilization; the assumption that the sterilization step leaves the graphene-oxide structure, the palladium chemical state, and the apatite morphology unchanged rests on the mildness of the procedure described in Section 2.6 rather than on a direct measurement.

## 4. Conclusions

A systematic series of HA, HA/Pd, HA/GO, and HA/GO/Pd coatings was hydrothermally deposited on β-Ti30Zr5Mo alloy to clarify how GO organization and Pd incorporation govern coating performance. XRD and FTIR confirmed preservation of the HA framework, while Raman spectroscopy verified retention of the GO-derived carbon phase in the GO-containing coatings. Detailed XPS analysis of HA/GO-2Pd further revealed oxygenated carbon environments and indicated that Pd was present predominantly in an oxidized and oxygen-coordinated state rather than as metallic Pd^0^.

HA-2Pd exhibited the most favorable potentiodynamic corrosion response among the investigated coatings, showing the lowest corrosion current density (1.46 ± 0.02 µA cm^−2^), the lowest corrosion rate (0.0127 ± 0.0002 mm year^−1^), and a mean total EIS resistance of 15.73 ± 0.13 kΩ cm^2^, close to that of pristine HA (16.52 ± 0.40 kΩ cm^2^). In contrast, HA/GO displayed the poorest barrier behavior, with a corrosion rate of 0.0366 ± 0.0006 mm year^−1^ and a mean total resistance of 8.50 ± 0.15 kΩ cm^2^. Thus, GO did not provide the conventional barrier enhancement expected from an ideally dispersed two-dimensional reinforcement under the present hydrothermal processing conditions.

The combined morphological, chemical, and electrochemical results support the composition-dependent mechanism summarized in Scheme 1. Folded GO domains together with plausible Ca^2+^-mediated restacking/aggregation and heterogeneous HA nucleation are proposed to have generated locally accessible electrolyte pathways that outweighed the potential tortuous-path benefit of GO. Incorporation of 1 wt% Pd partially restored coating coverage, increasing the mean total EIS resistance to 14.97 ± 0.20 kΩ cm^2^ and decreasing the corrosion rate to 0.0187 ± 0.0002 mm year^−1^. Increasing Pd to 2 wt% produced a different response: although the representative outer-layer resistance remained slightly higher, the representative charge-transfer resistance decreased from approximately 14.27 to 11.12 kΩ cm^2^, while the mean total resistance decreased to 11.73 ± 0.30 kΩ cm^2^ and the corrosion rate increased to 0.0253 ± 0.0005 mm year^−1^. These results indicate that the higher Pd loading altered the electrochemical behavior of buried GO–Pd–HA interfacial regions once electrolyte gained access to them rather than simply reducing outer-coating compactness.

Post-polarization observations further showed substantial retention of the HA-based coatings, indicating that localized electrolyte transport and interfacial processes, rather than catastrophic coating delamination, controlled the observed differences. The particularly low local Ca/P ratio of HA/GO-2Pd after polarization was also consistent with a composition-dependent alteration of the local dissolution/reprecipitation response, although the point-EDS values should be regarded as local and semi-quantitative rather than representative of bulk coating stoichiometry.

The biological responses did not follow the electrochemical ranking. All HA-based coatings supported greater short-term A7R5 cell densities than the uncoated β-Ti30Zr5Mo substrate, with HA/GO-2Pd showing the highest relative cell density (330.2% of control). However, the GO/Pd coatings simultaneously generated markedly higher *S. aureus* Xen36 bioluminescent responses than HA, HA/Pd, or HA/GO. The Xen36 results therefore indicate a greater bacterial reporter response rather than a direct CFU-derived increase in viable bacterial number. Among the investigated compositions, HA-2Pd provided the most balanced multifunctional response, combining the strongest corrosion protection, a high A7R5 cell-density response, and a comparatively low bacterial bioluminescent signal. Overall, the present findings demonstrate that optimization of HA/GO/Pd coatings requires control of GO organization, Pd-associated interfacial chemistry, electrolyte-access pathways, and buried charge-transfer processes rather than optimization of nominal additive content alone.

## Figures and Tables

**Figure 1:**
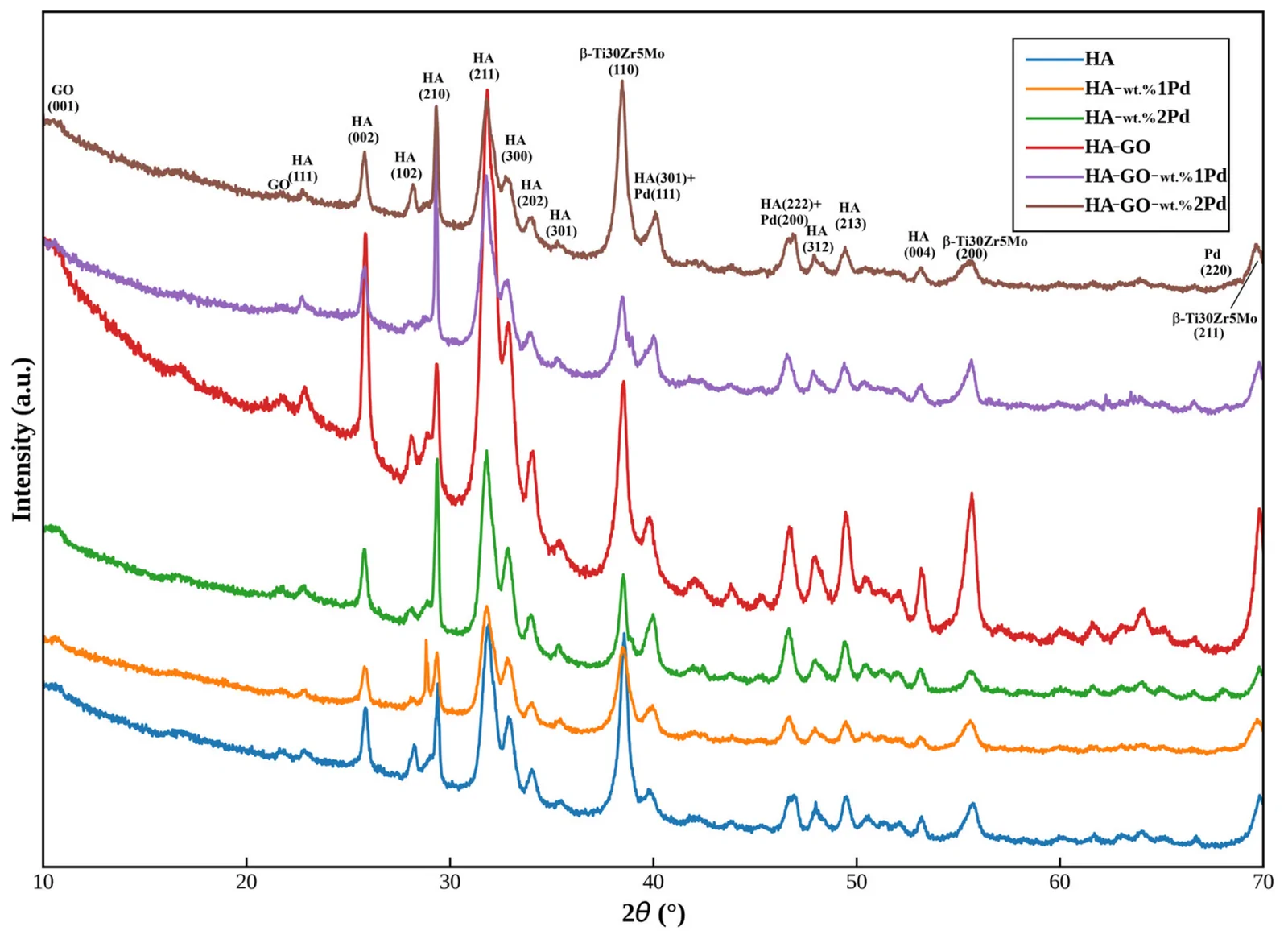
X-ray diffraction (XRD) patterns of the hydrothermally deposited HA, HA-1Pd, HA-2Pd, HA/GO, HA/GO-1Pd, and HA/GO-2Pd coatings on β-Ti30Zr5Mo alloy, showing the characteristic reflections of hydroxyapatite, the GO-associated low-angle diffraction feature, the weak diffraction contributions of the Pd-containing coatings, and the reflections of the β-Ti30Zr5Mo substrate. The patterns were acquired over 5–80° and are displayed over the diagnostically informative 10–70° interval, with no reflection having been detected outside it; they are shown as recorded and vertically offset for clarity, and every detected reflection is indexed in accordance with Table 2.

**Figure 2:**
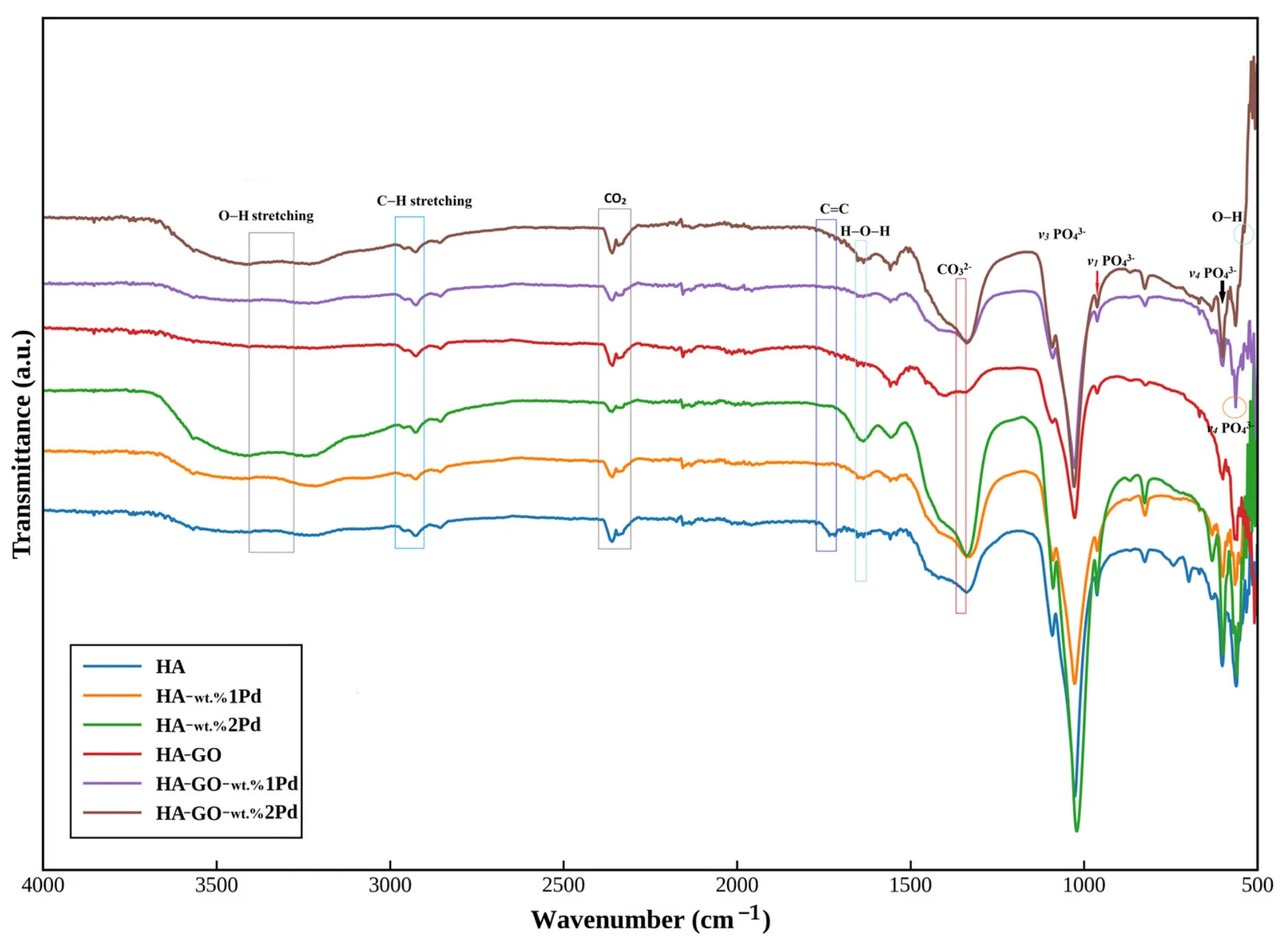
FTIR spectra of the hydrothermally deposited HA, HA-1Pd, HA-2Pd, HA/GO, HA/GO-1Pd, and HA/GO-2Pd coatings on β-Ti30Zr5Mo alloy, showing the characteristic phosphate, hydroxyl, carbonate-related, and C=C-associated vibrational features.

**Figure 3:**
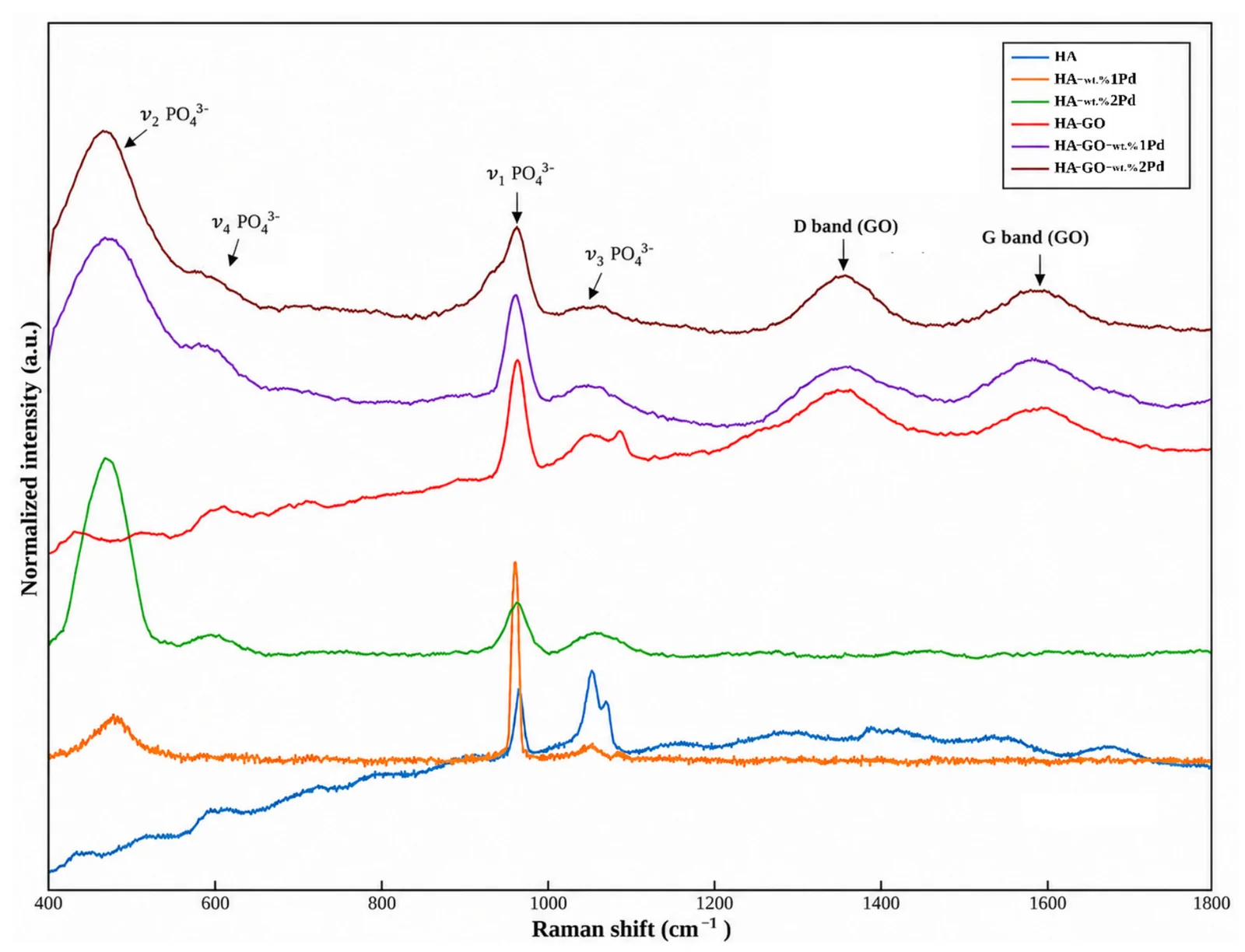
Raman spectra of the hydrothermally deposited HA, HA-1Pd, HA-2Pd, HA/GO, HA/GO-1Pd, and HA/GO-2Pd coatings on β-Ti30Zr5Mo alloy, showing the characteristic HA phosphate vibrations and the D and G bands associated with the GO-containing compositions.

**Figure 4:**
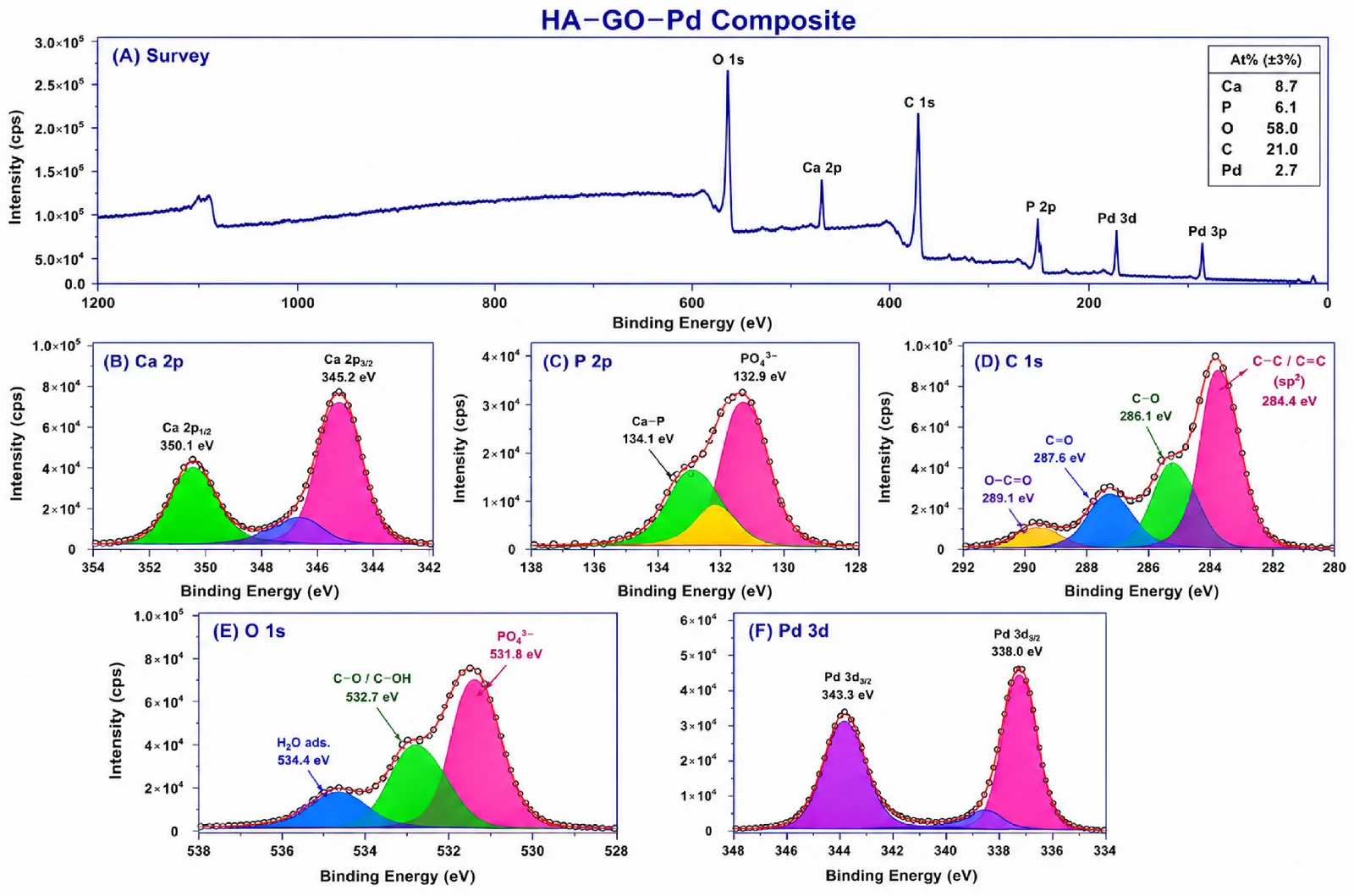
XPS analysis of the HA/GO-2Pd coating: (**A**) survey spectrum and high-resolution spectra of the (**B**) Ca 2p, (**C**) P 2p, (**D**) C 1s, (**E**) O 1s, and (**F**) Pd 3d regions.

**Figure 5:**
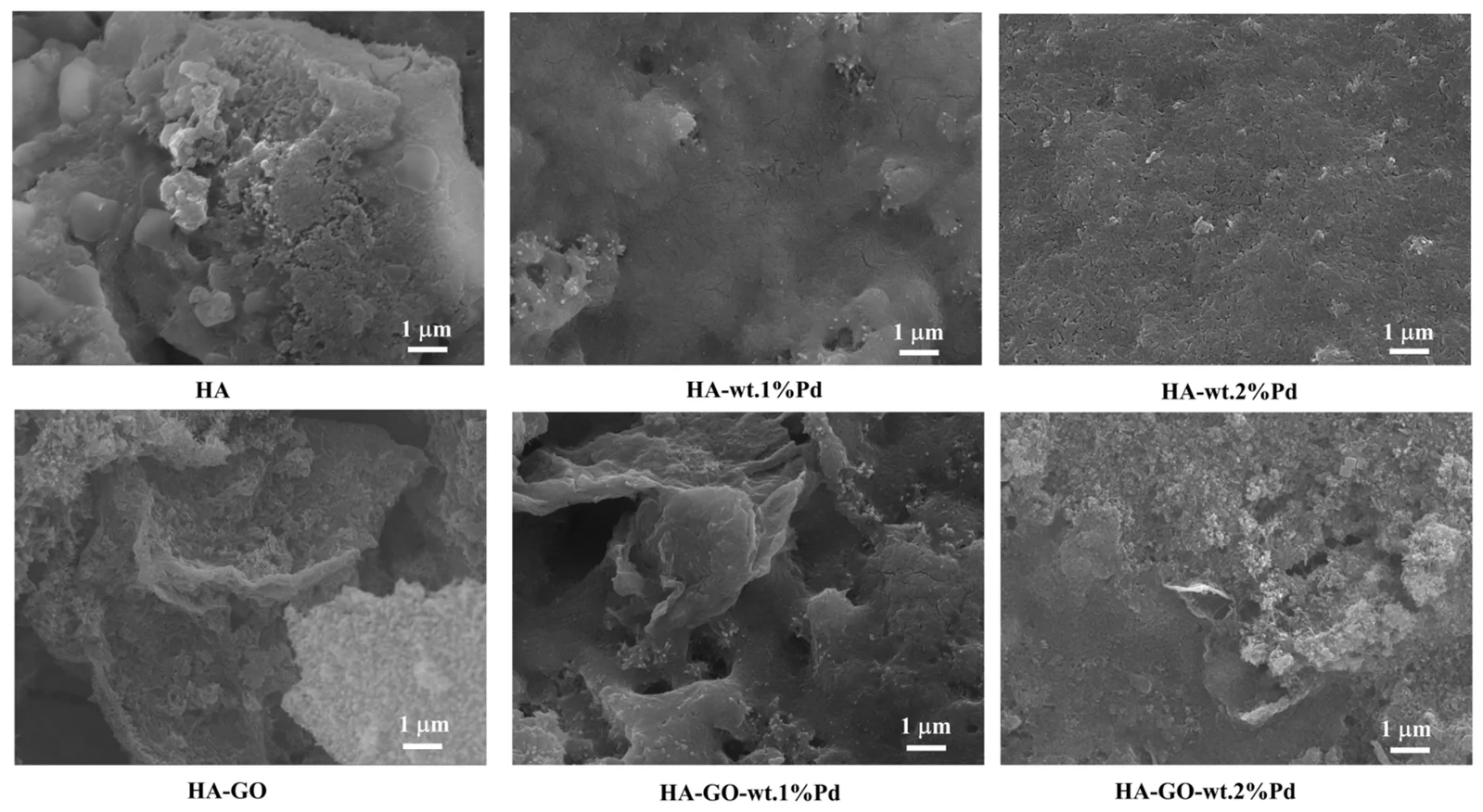
SEM micrographs of the as-deposited HA, HA-1Pd, HA-2Pd, HA/GO, HA/GO-1Pd, and HA/GO-2Pd coatings, illustrating composition-dependent changes in coating compactness, granular HA growth, and the folded sheet-like morphology associated with the GO-containing groups.

**Figure 6:**
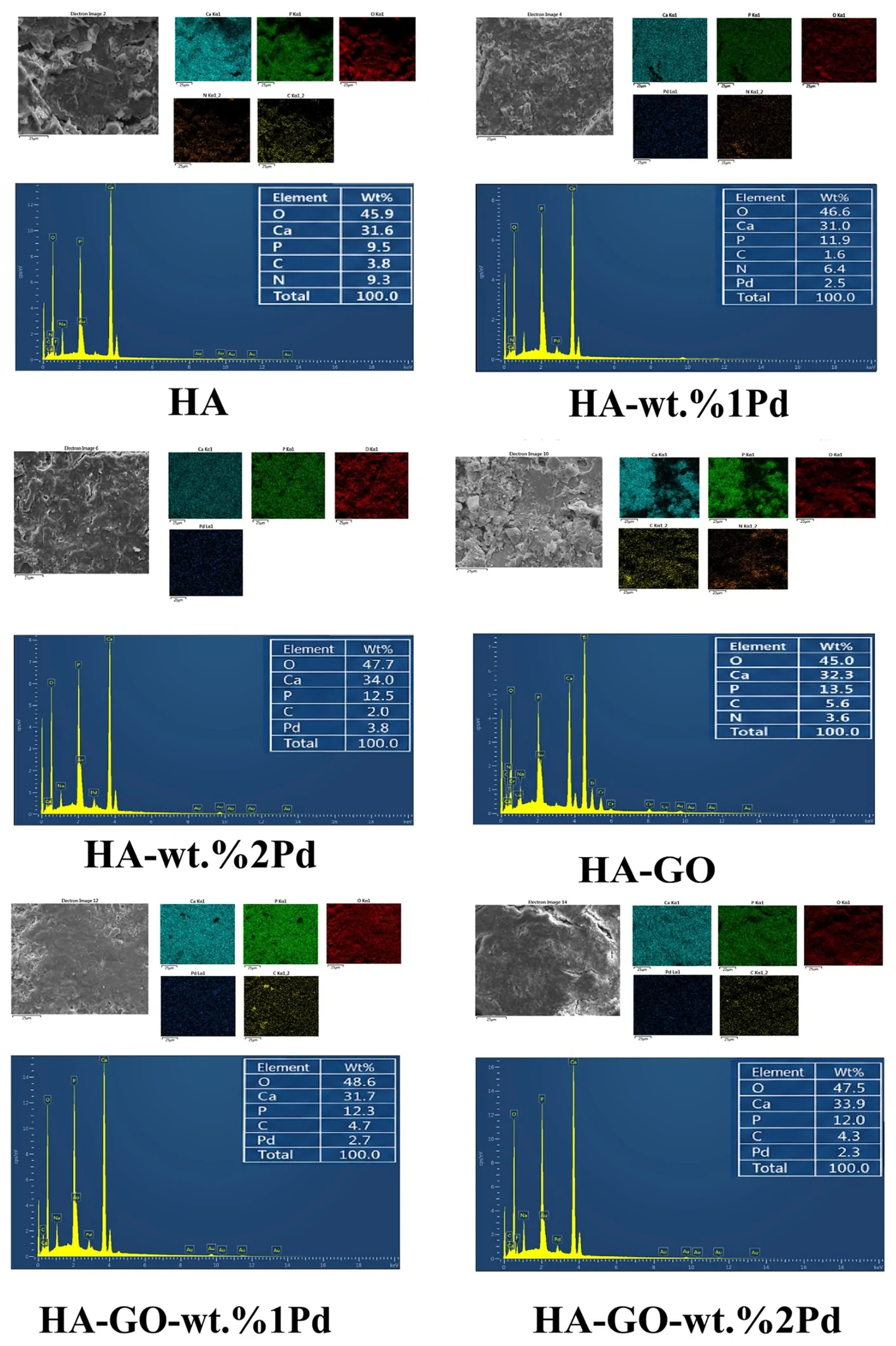
SEM–EDS elemental maps and corresponding spectra of the as-deposited coatings, showing the distribution of the principal coating elements and Pd together with the pronounced substrate-related contribution detected for HA/GO. The panels are arranged in two columns so that each map set and its corresponding map-sum spectrum are reproduced at a legible size.

**Figure 7:**
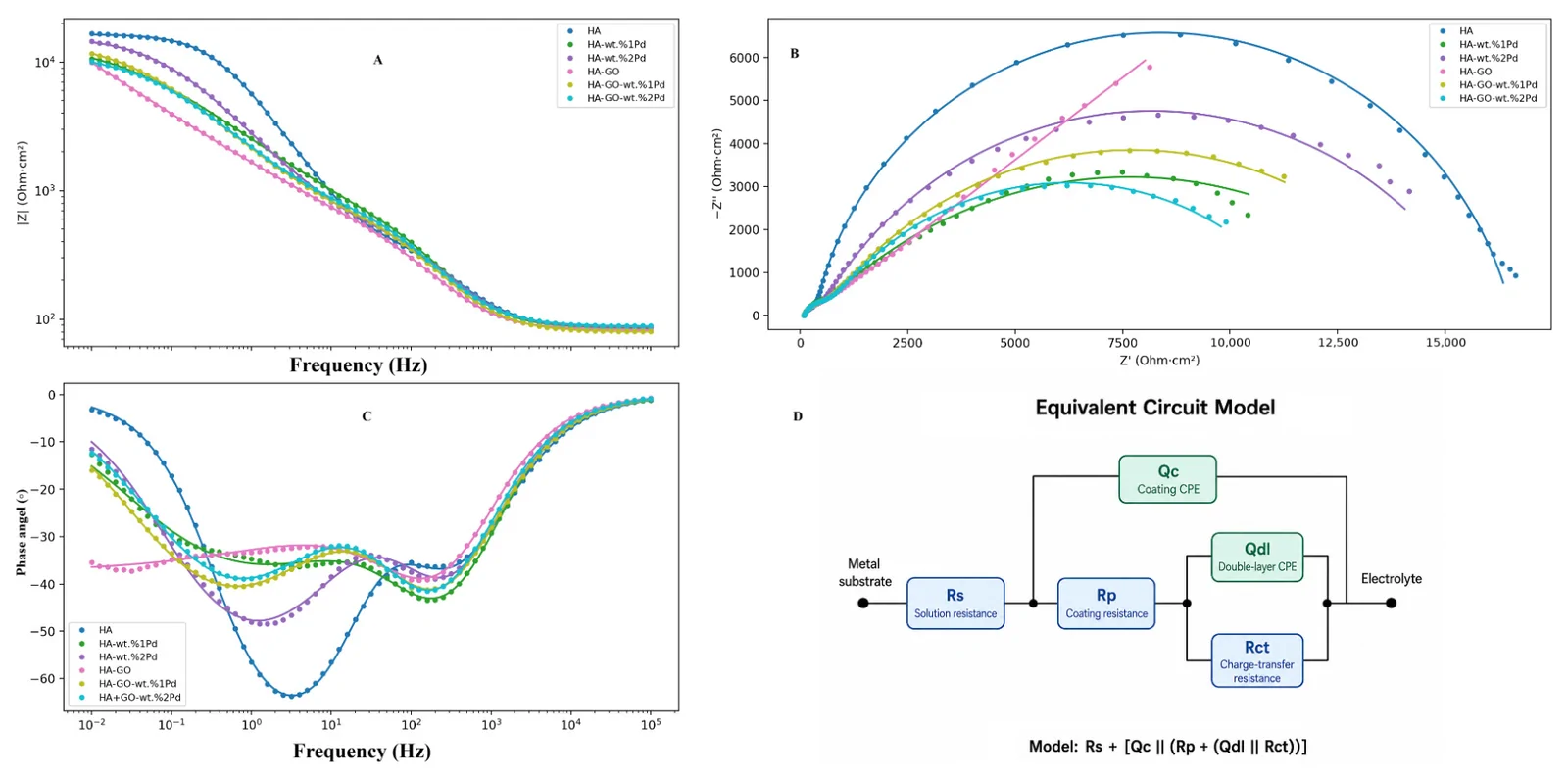
Electrochemical impedance behaviors of the HA, HA-1Pd, HA-2Pd, HA/GO, HA/GO-1Pd, and HA/GO-2Pd coatings in Hank’s solution at 37 °C: (**A**) Bode impedance modulus, (**B**) Nyquist plots, (**C**) Bode phase-angle plots, and (**D**) equivalent electrical circuit used for fitting. Symbols represent experimental data and solid lines represent the equivalent-circuit fits.

**Figure 8:**
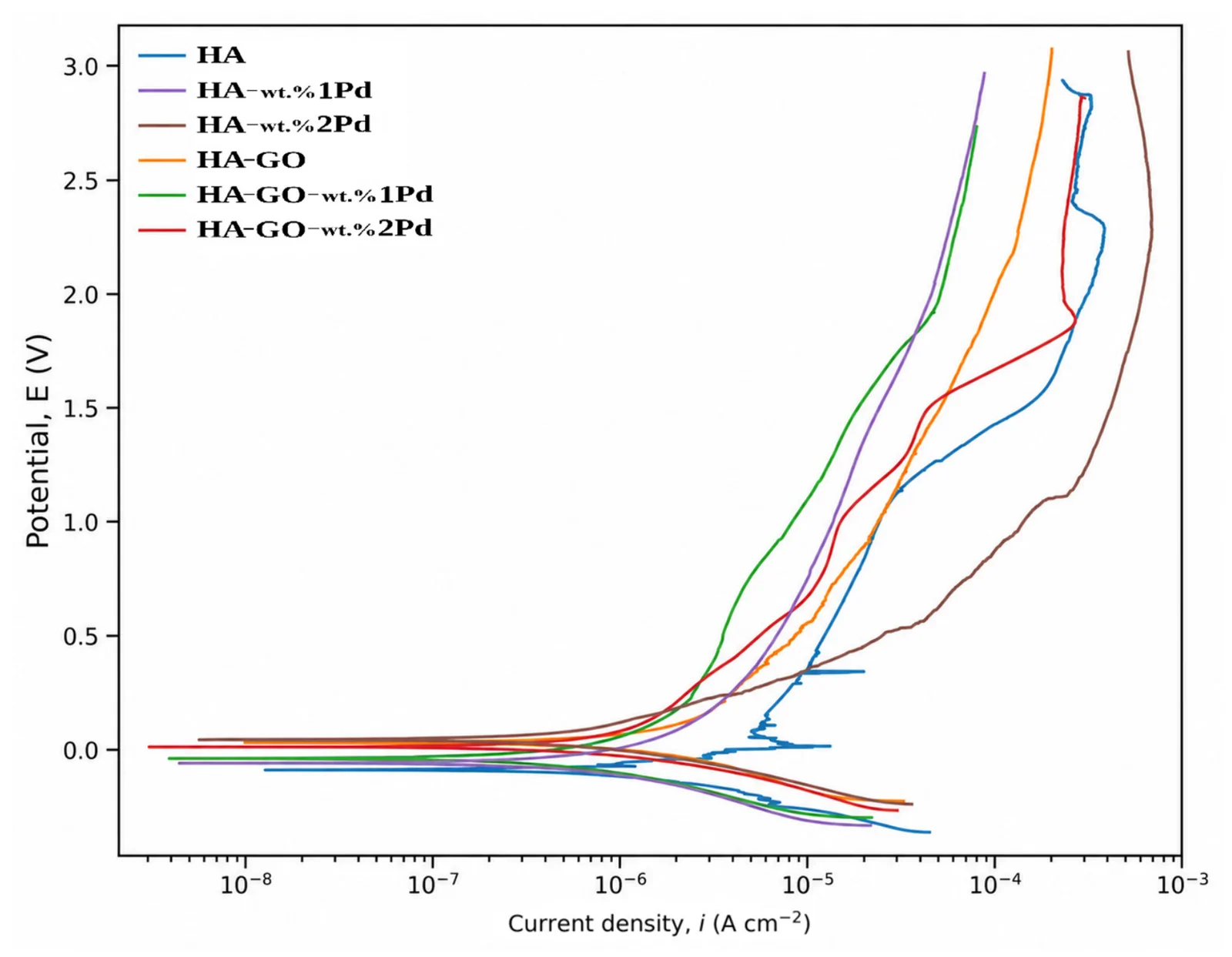
Potentiodynamic polarization curves of the HA, HA-1Pd, HA-2Pd, HA/GO, HA/GO-1Pd, and HA/GO-2Pd coatings recorded in Hank’s solution at 37 °C.

**Figure 9:**
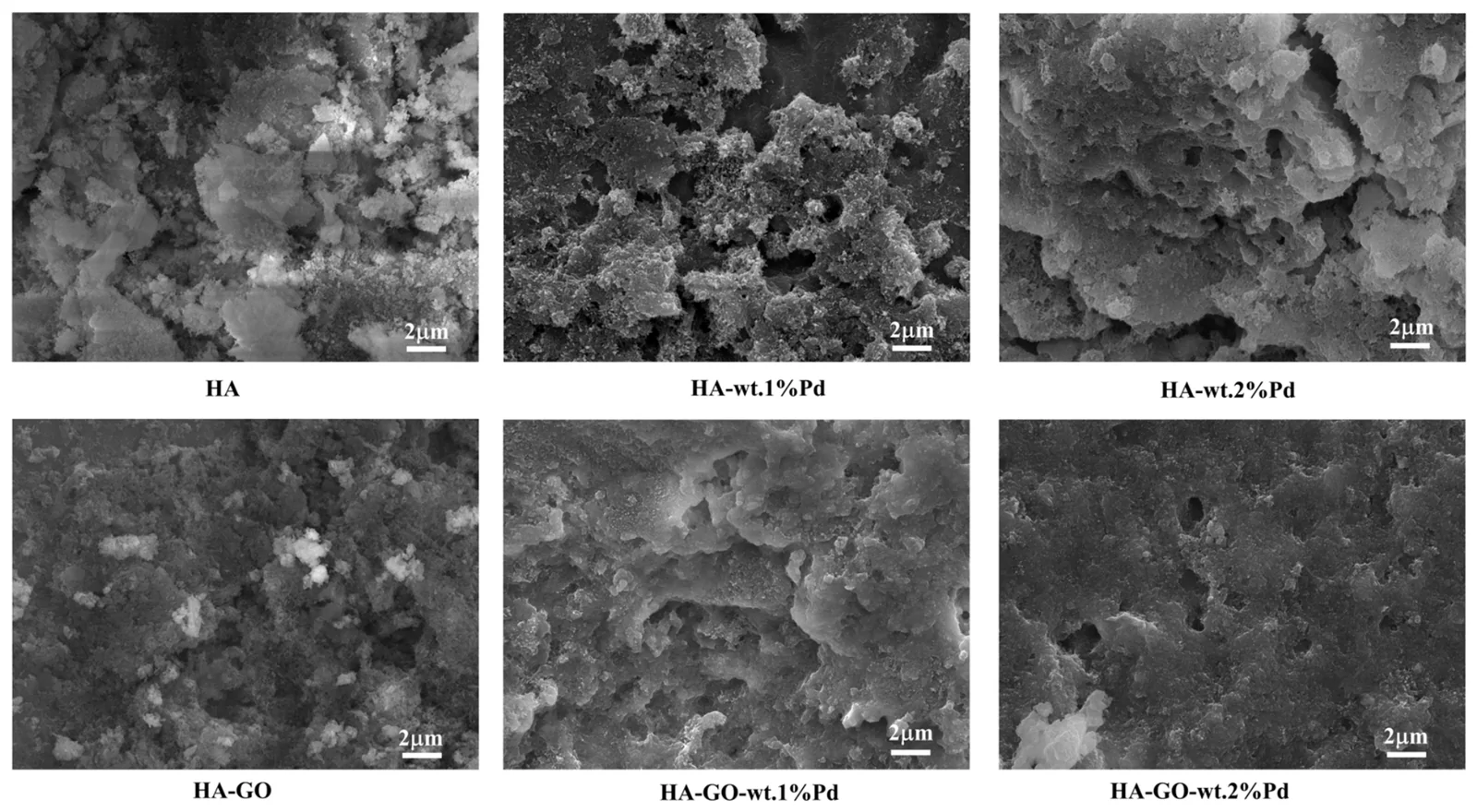
SEM micrographs of the HA, HA-1Pd, HA-2Pd, HA/GO, HA/GO-1Pd, and HA/GO-2Pd coating surfaces after potentiodynamic polarization testing, showing the retained coating morphologies following electrochemical exposure.

**Figure 10:**
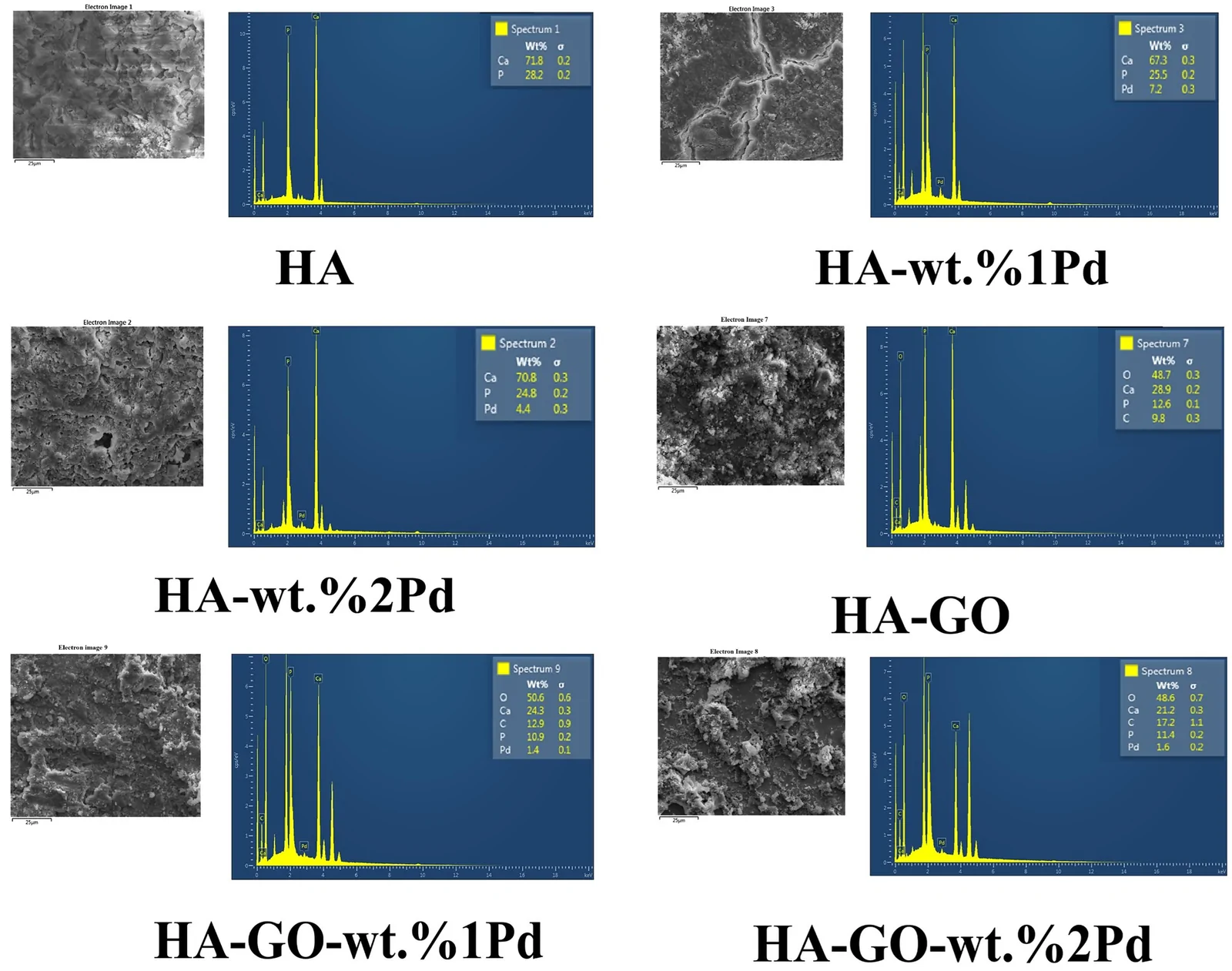
Point-EDS spectra acquired from the HA-based coating surfaces after potentiodynamic polarization testing, showing the retention and composition-dependent distribution of Ca, P, and Pd.

**Scheme 1:**
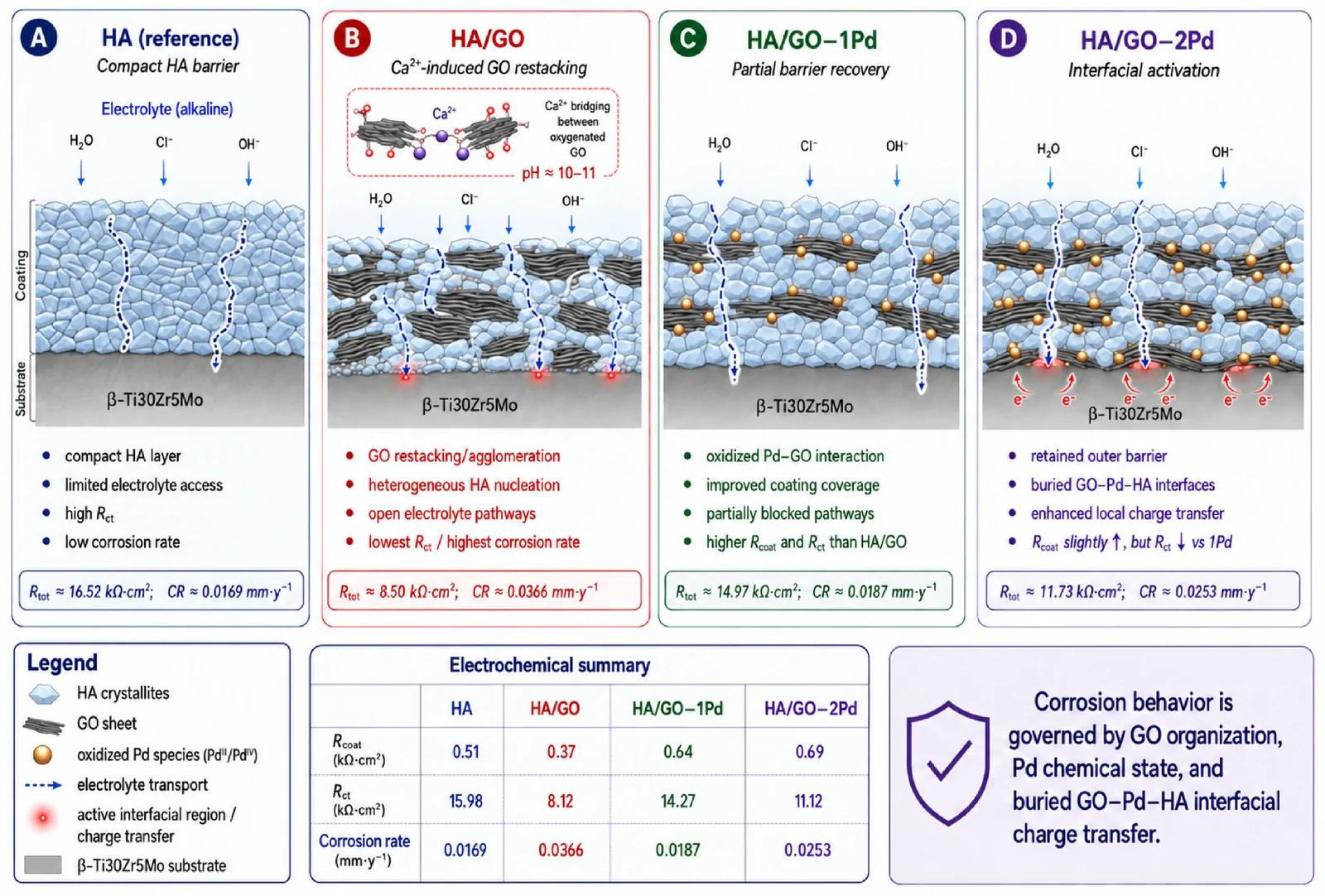
Proposed composition-dependent corrosion mechanism of the HA, HA/GO, HA/GO-1Pd, and HA/GO-2Pd coatings on β-Ti30Zr5Mo alloy. (**A**) HA reference coating, showing a comparatively compact HA barrier with limited electrolyte access and low interfacial charge transfer. (**B**) HA/GO coating, illustrating proposed Ca^2+^-mediated GO restacking/aggregation, heterogeneous HA nucleation, and open electrolyte-transport pathways leading to increased access to the coating/substrate interface. (**C**) HA/GO-1Pd coating, showing partial recovery of coating coverage associated with oxidized Pd–GO interactions and partial blocking of electrolyte pathways. (**D**) HA/GO-2Pd coating, illustrating retention of the outer coating barrier together with increased electrochemical activity at buried GO–Pd–HA interfacial regions. **Blue downward arrows indicate electrolyte penetration/transport toward the coating/substrate interface, whereas red arrows indicate interfacial electron/charge-transfer processes at electrochemically active regions.** The scheme illustrates the proposed effects of GO organization, Pd-associated interfacial chemistry, electrolyte accessibility, and buried charge-transfer processes on the measured corrosion response.

**Figure 11:**
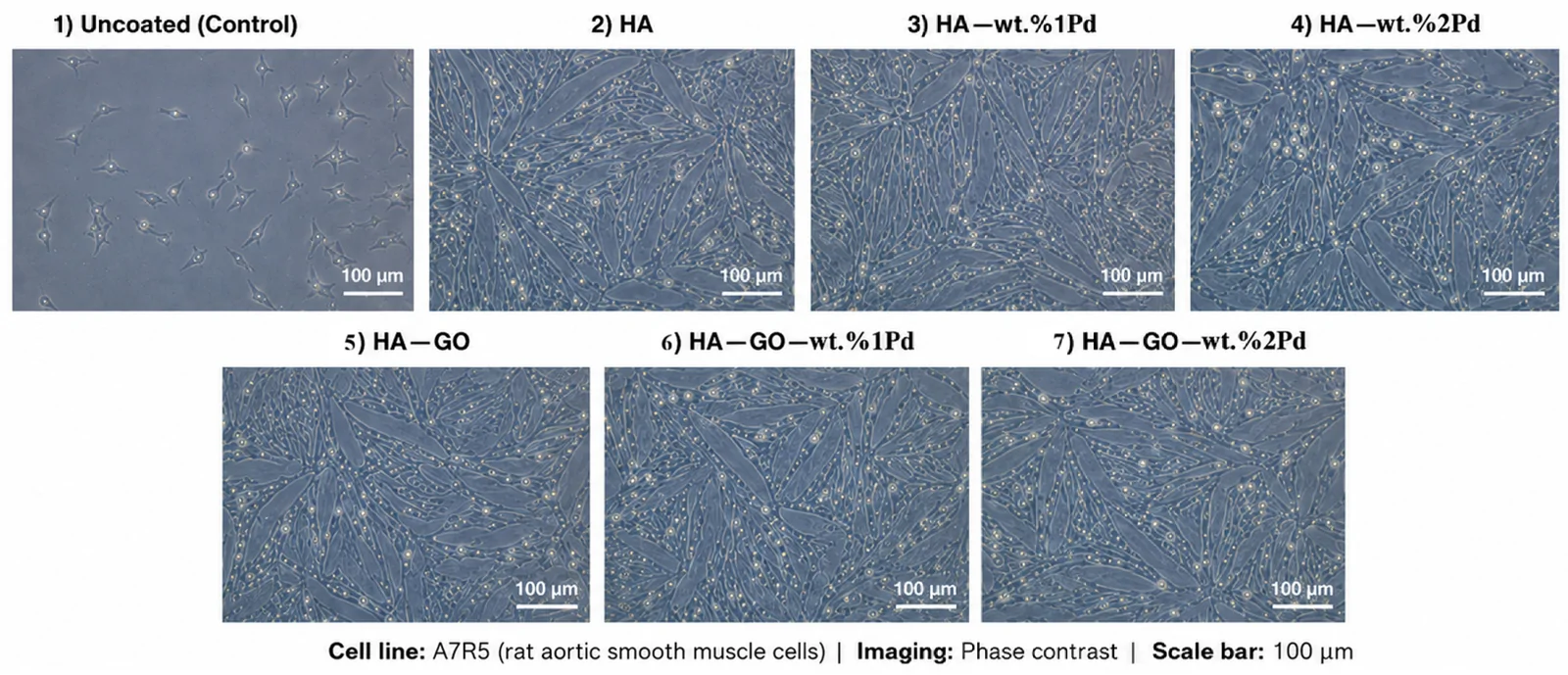
Phase-contrast micrographs of A7R5 cells cultured for 24 h on the uncoated β-Ti30Zr5Mo control and on the HA, HA-1Pd, HA-2Pd, HA/GO, HA/GO-1Pd, and HA/GO-2Pd coatings. Scale bar: 100 µm.

**Figure 12:**
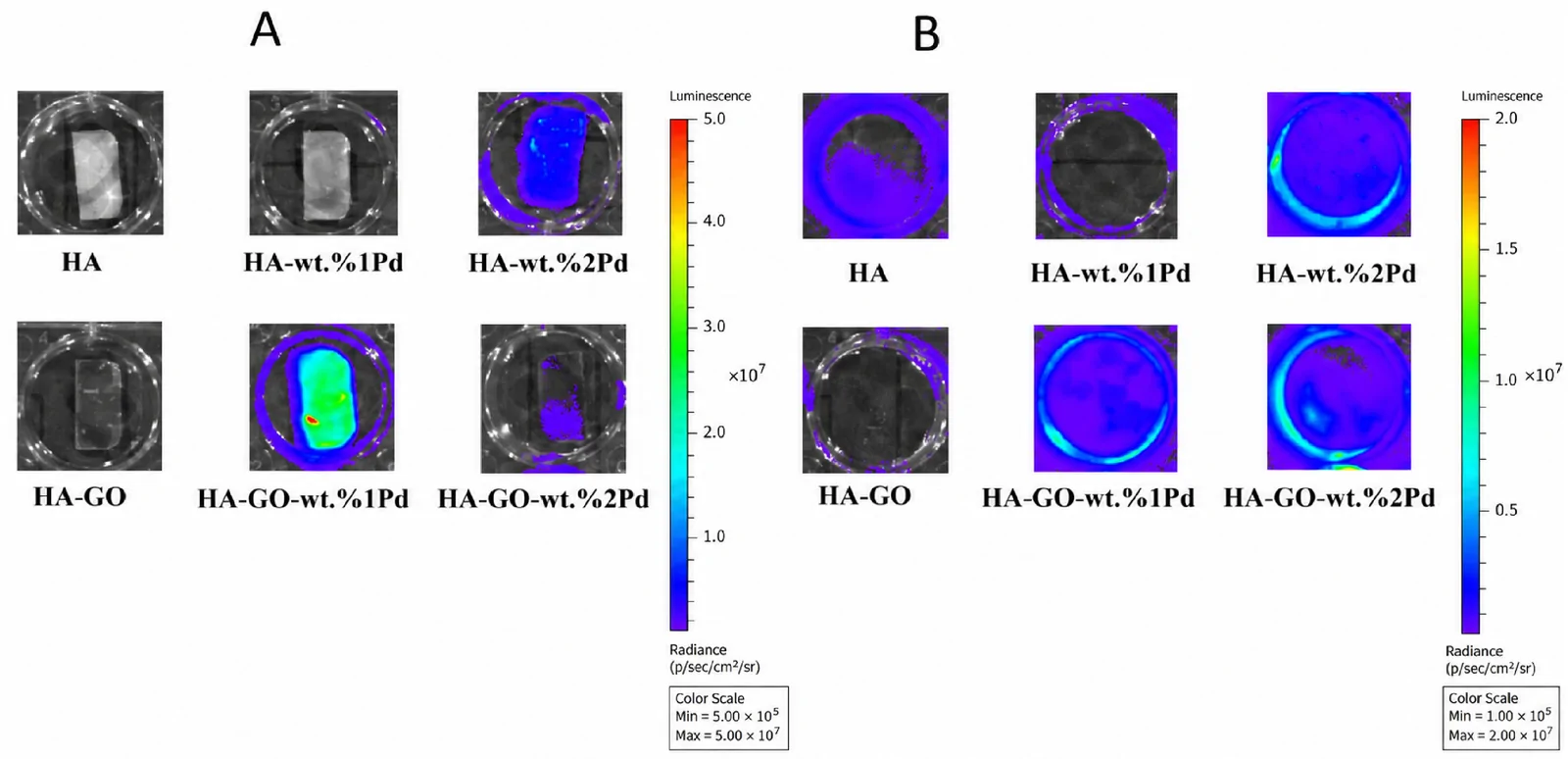
IVIS bioluminescence images of Staphylococcus aureus Xen36 following incubation with the HA, HA-1Pd, HA-2Pd, HA/GO, HA/GO-1Pd, and HA/GO-2Pd coatings: (A) bioluminescence images recorded in the presence of the coated specimens; (B) corresponding bioluminescence images acquired after removal of the specimens from the wells, showing the residual bacterial bioluminescence in the surrounding medium/well surface. Because different color-scale ranges were used for visualization, quantitative intergroup comparisons were based on the total radiant flux values shown in Figure 13.

**Figure 13:**
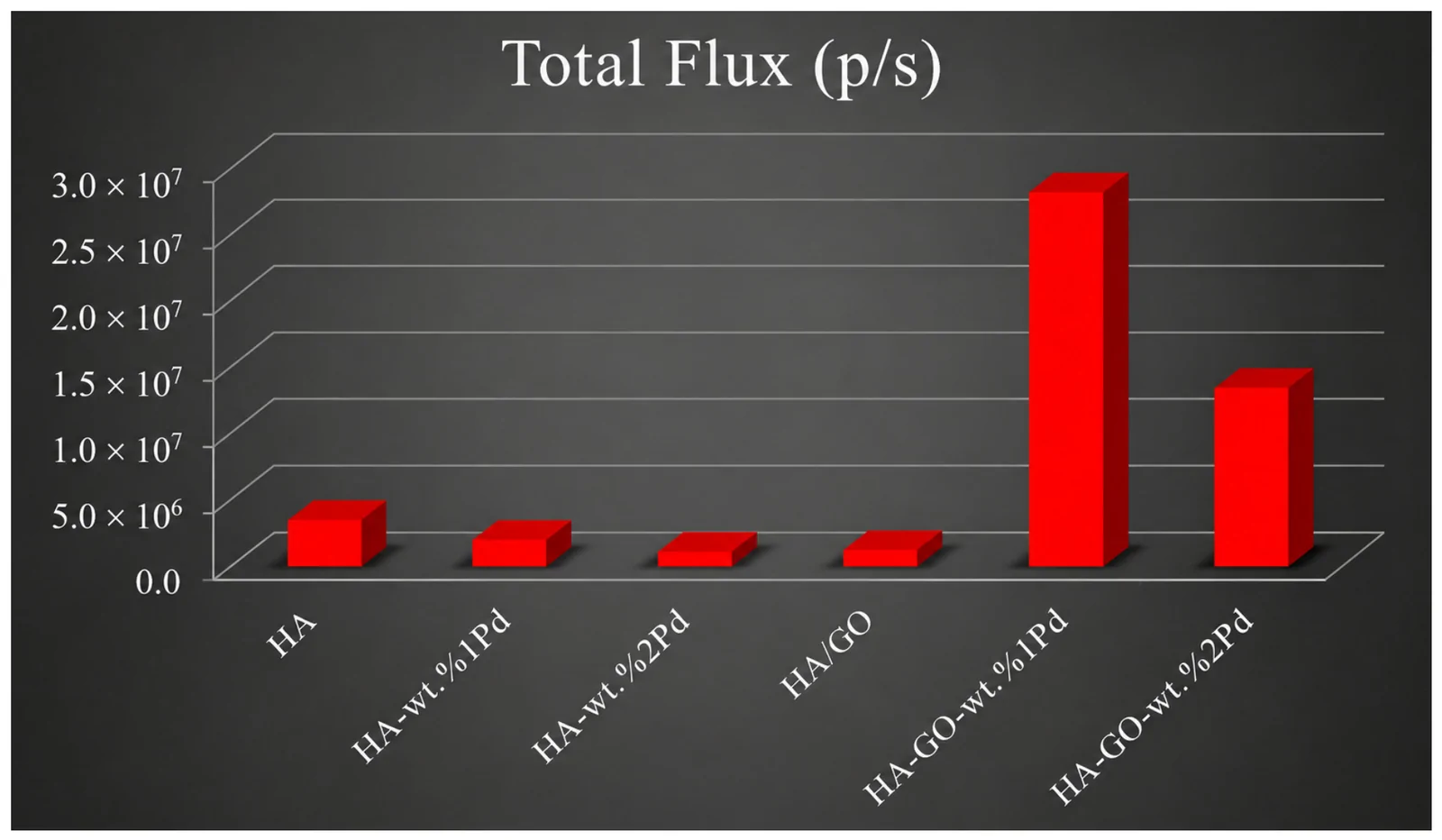
Total radiant flux of *Staphylococcus aureus* Xen36 associated with the six HA-based coating compositions, expressed as photons/s. Higher radiant flux represents a greater bacterial bioluminescent response under the standardized assay conditions.

**Table 1:** Nominal compositions and sample codes of the hydrothermally deposited coatings.

Sample	GO (wt%, Ca + P Mass Basis)	Pd (wt%, Elemental; Ca + P Mass Basis)
HA	–	–
HA-1Pd	–	1
HA-2Pd	–	2
HA/GO	1	–
HA/GO-1Pd	1	1
HA/GO-2Pd	1	2

**Table 2:** Indexing of all reflections detected in the grazing-incidence X-ray diffraction patterns of Figure 1.

2θ (°)	Phase	Plane	Comment
10–12	Graphene oxide	(001)	Present only in the GO-containing coatings; expanded interlayer spacing
21.8/22.9	Hydroxyapatite	(200)/(111)	Overlapping; the broad GO-related carbon contribution cannot be separated from these apatite reflections
25.9	Hydroxyapatite	(002)	c-axis texture indicator
28.1/29.0	Hydroxyapatite	(102)/(210)	–
31.8/32.2/32.9	Hydroxyapatite	(211)/(112)/(300)	Dominant apatite envelope
34.0/35.5	Hydroxyapatite	(202)/(301)	–
38.5	β-Ti30Zr5Mo	(110)	Strongest substrate reflection
39.8/40.1	Hydroxyapatite/Pd	(310)/(111)	Overlapping; Pd only in Pd-containing coatings
42.0/43.8/45.3	Hydroxyapatite	(311)/(113)/(203)	Newly indexed; enhanced in HA/GO
46.7	Hydroxyapatite + Pd	(222) + (200)	Overlapping reflections
48.1/49.5/50.5	Hydroxyapatite	(312)/(213)/(321)	–
52.1/53.1	Hydroxyapatite	(402)/(004)	Newly indexed; second texture indicator
55.5	β-Ti30Zr5Mo	(200)	Substrate; most intense for HA/GO
61–65	Hydroxyapatite	(511)/(304) group	Expected here but not resolved above the background in the present patterns
≈68	Pd	(220)	Overlaps the β-(211) reflection; not resolved as an independent peak
≈69.7	β-Ti30Zr5Mo	(211)	Second-strongest substrate reflection; at the right-hand edge of the displayed range

**Table 3:** FTIR band assignments for the HA, Pd-modified HA, and GO-containing HA coatings.

Wavenumber (cm^−1^)	Vibrational Mode	Assignment	Main Contribution	Interpretation in the Present Coatings
3200–3400	ν(O–H)	O–H stretching	HA/adsorbed H_2_O/GO	Broad band observed in all coatings; GO-containing groups may additionally contribute through residual hydroxyl functionalities
2340–2360	—	Atmospheric CO_2_	Artifact	Weak atmospheric interference; not assigned to the coating chemistry
2850–2960	ν(C–H)	C–H stretching	Residual organics/GO	Weak; aliphatic groups associated with the graphene-oxide surface and residual organic species
2000–2200	—	Combination and overtone modes of PO_4_^3−^	HA	Very weak and broad; no chemical modification implied
1630–1650	δ(H–O–H)	Adsorbed-water bending	HA/adsorbed H_2_O	Present throughout the coating series and partially overlaps with the GO-related C=C contribution
~1580–1625	ν(C=C)	Skeletal vibration of sp^2^-hybridized carbon domains	GO	Discernible/enhanced in HA/GO, HA/GO-1Pd, and HA/GO-2Pd; provides supporting evidence for retention of the GO-derived carbon framework
1716–1733	ν(C=O)	Carbonyl/carboxyl groups	GO	Weak or insufficiently resolved as an independent feature; may be affected by the relatively low GO content and surrounding HA-related absorptions
1337–1400	ν_3_(CO_3_^2−^)	Carbonate-related vibration	HA	Present throughout the coating series; shifted toward ~1399 cm^−1^ for HA/GO compared with ~1337–1339 cm^−1^ for most Pd-containing coatings
1225–1266	ν(C–O)	Epoxide/C–O functionality	GO	Located on the shoulder of the intense phosphate region and not reliably resolved as an isolated band
1020–1092	ν_3_(PO_4_^3−^)	Asymmetric phosphate stretching	HA	Dominant FTIR band in all coatings; GO C–O/alkoxy contributions around 1038–1054 cm^−1^ may overlap with this envelope
962–963	ν_1_(PO_4_^3−^)	Symmetric phosphate stretching	HA	Characteristic HA vibration observed as a shoulder of the main phosphate envelope
600–630	ν_4_(PO_4_^3−^)	Phosphate bending	HA	Characteristic HA bending vibration retained throughout the coating series
559–565	ν_4_(PO_4_^3−^)	Phosphate bending	HA	Second component of the characteristic phosphate bending doublet

**Table 4:** Equivalent-circuit parameters obtained from representative EIS fits using the Rs(Qc(Rcoat(Qdl·Rct))) model, together with the mean total resistance values determined from three independently prepared specimens.

Sample	Rs (Ω·cm^2^)	Rcoat (Ω·cm^2^)	Rct (Ω·cm^2^)	Qdl (Ω^−1^·s^n^)	ndl	Rtot = Rcoat + Rct (kΩ·cm^2^)	χ^2^
HA	84.51	513.9	15,977.8	1.916 × 10^−5^	0.915	16.52 ± 0.40	3.34 × 10^−5^
HA-1Pd	85.27	582.8	14,300.0	1.525 × 10^−4^	0.524	14.79 ± 0.37	1.23 × 10^−4^
HA-2Pd	86.79	502.1	15,385.2	9.130 × 10^−5^	0.687	15.73 ± 0.13	1.19 × 10^−4^
HA/GO	82.38	368.2	8122.0	3.249 × 10^−4^	0.417	8.50 ± 0.15	5.09 × 10^−5^
HA/GO-1Pd	79.27	638.4	14,268.0	1.581 × 10^−4^	0.612	14.97 ± 0.20	1.25 × 10^−5^
HA/GO-2Pd	87.81	686.2	11,120.2	1.528 × 10^−4^	0.626	11.73 ± 0.30	1.74 × 10^−5^

Note: Equivalent-circuit parameters are reported from representative EIS fits, whereas Rtot values are presented as mean ± standard deviation from three independently prepared specimens (n = 3). Rtot was calculated as Rcoat + Rct for each specimen.

**Table 5:** Constant-phase-element parameters associated with the outer-coating layer obtained from EIS fitting.

Sample	Qc (Ω^−1^·s^n^)	nc
**HA**	1.365 × 10^−5^	0.790
**HA-1Pd**	9.981 × 10^−6^	0.833
**HA-2Pd**	1.099 × 10^−5^	0.823
**HA/GO**	1.540 × 10^−5^	0.814
**HA/GO-1Pd**	1.526 × 10^−5^	0.795
**HA/GO-2Pd**	1.361 × 10^−5^	0.807

**Table 6:** Electrochemical parameters derived from potentiodynamic polarization measurements of the coating series. Values are presented as mean ± standard deviation (SD) from three independently prepared specimens (n = 3).

Sample	Ecorr (V)	Icorr (µA/cm^2^)	βa (mV/dec)	βc (mV/dec)	Rp (kΩ·cm^2^)	Corrosion Rate (mm/yr)
**HA**	−0.0856 ± 0.0032	1.95 ± 0.04	88.2 ± 2.2	100.1 ± 1.9	10.44 ± 0.21	0.0169 ± 0.0003
**HA-1Pd**	−0.0583 ± 0.0021	3.14 ± 0.07	405.0 ± 11.1	248.8 ± 5.7	21.29 ± 0.56	0.0273 ± 0.0006
**HA-2Pd**	+0.0438 ± 0.0015	1.46 ± 0.02	196.0 ± 1.5	116.7 ± 2.0	21.79 ± 0.21	0.0127 ± 0.0002
**HA/GO**	+0.0321 ± 0.0022	4.21 ± 0.07	324.5 ± 6.7	210.8 ± 4.7	13.19 ± 0.15	0.0366 ± 0.0006
**HA/GO-1Pd**	−0.0373 ± 0.0018	2.15 ± 0.04	383.3 ± 6.1	209.2 ± 1.8	27.37 ± 0.37	0.0187 ± 0.0002
**HA/GO-2Pd**	+0.0124 ± 0.0020	2.91 ± 0.06	477.8 ± 11.7	161.8 ± 4.1	18.02 ± 0.44	0.0253 ± 0.0005

**Table 7:** Post-polarization surface compositions determined by point EDS and calculated local Ca/P atomic ratios. The measurements are semi-quantitative and represent local near-surface regions after electrochemical exposure.

Sample	Ca (wt%)	P (wt%)	Pd (wt%)	Ca/P (At., Calc.)
HA	71.8	28.2	–	1.97
HA-1Pd	67.3	25.5	7.2	2.04
HA-2Pd	70.8	24.8	4.4	2.21
HA/GO	28.9	12.6	–	1.77
HA/GO-1Pd	24.3	10.9	1.4	1.72
HA/GO-2Pd	21.2	11.4	1.6	1.44

**Table 8:** A7R5 cell density after 24 h of culture and relative cell density normalized to the uncoated β-Ti30Zr5Mo control. Cell-density values are presented as mean ± standard deviation (SD) from three independently prepared specimens (n = 3).

Sample	Cell Density (Cells/cm^2^), Mean ± SD	Relative Cell Density (% of Control)
Uncoated β-Ti30Zr5Mo (control)	6502 ± 175	100.0
HA	16,058 ± 420	247.0
HA-1Pd	19,107 ± 503	293.9
HA-2Pd	20,513 ± 501	315.5
HA/GO	18,986 ± 390	292.0
HA/GO-1Pd	20,025 ± 458	308.0
HA/GO-2Pd	21,471 ± 516	330.2

## Data Availability

The data that support the findings of this study are available from the corresponding author upon reasonable request.
